# Adeno‐Associated Virus 8 and 9 Myofibre Type/Size Tropism Profiling Reveals Therapeutic Effect of Microdystrophin in Canines

**DOI:** 10.1002/jcsm.13681

**Published:** 2025-01-10

**Authors:** Matthew J. Burke, Braiden M. Blatt, James A. Teixeira, Dennis O. Pérez‐López, Yongping Yue, Xiufang Pan, Chady H. Hakim, Gang Yao, Roland W. Herzog, Dongsheng Duan

**Affiliations:** ^1^ Department of Molecular Microbiology and Immunology, School of Medicine University of Missouri Columbia Missouri USA; ^2^ College of Veterinary Medicine University of Missouri Columbia Missouri USA; ^3^ Department of Chemical and Biomedical Engineering, College of Engineering University of Missouri Columbia Missouri USA; ^4^ Department of Pediatrics, Herman B Wells Center for Pediatric Research Indiana University Indianapolis Indiana USA; ^5^ Department of Biomedical Sciences, College of Veterinary Medicine University of Missouri Columbia Missouri USA; ^6^ Department of Neurology, School of Medicine University of Missouri Columbia Missouri USA

**Keywords:** adeno‐associated virus (AAV), canine model, Duchenne muscular dystrophy (DMD), microdystrophin, myofibre size, myofibre type

## Abstract

**Background:**

Adeno‐associated virus (AAV) 8 and 9 are in clinical trials for treating neuromuscular diseases such as Duchenne muscular dystrophy (DMD). Muscle consists of myofibres of different types and sizes. However, little is known about the fibre type and fibre size tropism of AAV in large mammals.

**Methods:**

We evaluated fibre type‐ and size‐specific transduction properties of AAV8 and AAV9 in 17 dogs that received systemic gene transfer (dose 1.94 ± 0.52 × 10^14^ vg/kg; injected at 2.86 ± 0.30 months; harvested at 20.79 ± 3.30 months). For AAV8, two DMD dogs and three carrier dogs received an alkaline phosphatase (AP) reporter vector, and five DMD dogs received a four‐repeat microdystrophin (uDys) vector. For AAV9, one normal and one DMD dog received the AP vector, and five DMD dogs received a five‐repeat uDys vector. Association between AAV transduction and the fibre type/size was studied in three muscles that showed mosaic transgene expression, including the biceps femoris, teres major and latissimus dorsi.

**Results:**

Transgene expression was detected in 30%–45% of myofibres. In the AP reporter vector–injected dogs, neither AAV8 nor AAV9 showed a statistically significant fibre type preference. Interestingly, AP expression was enriched in smaller fibres. In uDys‐treated DMD dogs, slow and fast myofibres were equally transduced. Notably, uDys‐expressing myofibres were significantly larger than uDys‐negative myofibres irrespective of the AAV serotype (*p* < 0.0001). In AAV8 uDys vector–injected dogs, the mini‐Feret diameter was 15%, 16% and 23% larger in uDys‐positive slow, fast and hybrid fibres, respectively; the cross‐sectional area was 30%, 34% and 46% larger in uDys‐positive slow, fast and hybrid fibres, respectively. In AAV9 uDys vector–injected dogs, the mini‐Feret diameter was 12%, 13% and 25% larger in uDys‐positive slow, fast and hybrid fibres, respectively; the cross‐sectional area was 25%, 28% and 59% larger in uDys‐positive slow, fast and hybrid fibres, respectively.

**Conclusions:**

Our studies suggest that AAV8 and AAV9 transduce fast and slow myofibres at equivalent efficiency. Importantly, uDys therapy effectively prevented dystrophic myofibre atrophy. Our study provides important insight into systemic muscle AAV delivery in large mammals and supports further development of uDys gene therapy for DMD.

## 1 Introduction

Adeno‐associated virus (AAV) is a small, non‐enveloped, single‐strand DNA virus. AAV vectors (such as AAV serotypes 8 and 9) have been extensively used to treat inherited neuromuscular diseases, including spinal muscular atrophy, myotubular myopathy, limb‐girdle muscular dystrophy and Duchenne muscular dystrophy (DMD). The success of AAV gene therapy is highly dependent on the tissue and cell‐type specificity of the AAV capsids [1].

Skeletal muscle comprises myofibres of distinctive phenotypes, including Type I, Type II and hybrid myofibres [2, 3]. Type I fibres express slow myosin heavy chain. Type II fibres express fast myosin heavy chain. Myofibres that express both slow and fast myosin heavy chains are referred to as hybrid fibres. Type II fibres can be further divided into Types IIa, IIb and IIx. Type I fibres are slow oxidative fibres, and they generate smaller force but are resistant to fatigue. Type IIa fibres are fast oxidative/glycolytic fibres, and they generate moderately high force at a relatively fast rate and are moderately resistant to fatigue. Type IIb fibres are fast glycolytic fibres, and they generate very high force at a very fast rate but fatigue very quickly. Type IIx fibres are also fast glycolytic fibres but exhibit a contractile profile intermediate between Type IIa and IIb fibres.

Myofibre type preference of AAV vectors has been extensively studied in murine models [4, 5, 6, 7, 8, 9, 10, 11, 12]. However, mouse skeletal muscle is enriched with Type IIb fibres, which are undetectable in most human muscles [13]. Trunk and limb muscles in dogs more accurately reflect the fibre type composition of human muscles [14, 15].

Little is known about the fibre type preference of AAV in large mammals. To address this issue, we analysed muscles from 17 dogs that received systemic injections of AAV8 or AAV9, two serotypes widely used in systemic gene therapy studies. These AAV vectors expressed either alkaline phosphatase (AP) reporter or therapeutic microdystrophin (uDys). The AP vectors were delivered to normal, carrier or DMD dogs. The uDys vectors were only delivered to DMD dogs. We found AAV8 and AAV9 transduced slow and fast myofibres at similar efficiency. Importantly, uDys significantly prevented myofibre atrophy in dystrophic dogs.

## 2 Methods

### 2.1 Experimental Animals

All animal experiments were approved by the Animal Care and Use Committee of the University of Missouri. All experimental dogs were on a mixed genetic background. There were three different *DMD* gene mutations in affected dogs, including a point mutation in intron 6, a repetitive element‐1 insertion in intron 13 and a repetitive element insertion in intron 19. The *DMD* null mutations were genotyped by the polymerase chain reaction according to our published protocols [16, 17]. The age, sex and genotype of the experimental dogs are shown in Table 1. All dogs were negative for AAV8 and AAV9 neutralizing antibodies before AAV injection.

**TABLE 1:** Experimental dogs and AAV delivery.

Dog ID	Genotype	Diagnosis	Sex	AAV serotype	Transgene	AAV dose (vg/kg)	Immune suppression	Age at injection (m)	Age at harvest (m)	Study duration (m)
Dog #1	GL	Affected	F	AAV8	AP	1.07E + 14	CsA/MMF	4.53	7.27	2.73
Dog #2	GW	Affected	F	AAV8	AP	1.02E + 14	None	4.30	6.93	2.63
Dog #3	GX	Carrier	F	AAV8	AP	1.03E + 14	None	3.43	18.13	14.70
Dog #4	GX	Carrier	F	AAV8	AP	2.52E + 14	None	0.07	12.50	12.43
Dog #5	GX	Carrier	F	AAV8	AP	8.76E + 13	CsA/MMF	3.70	18.17	14.47
Dog #6	LY	Affected	M	AAV8	4R uDys	1.00E + 14	Prednisolone	3.47	14.17	10.70
Dog #7	GW	Affected	F	AAV8	4R uDys	1.00E + 14	Prednisolone	3.27	14.07	10.80
Dog #8	GL	Affected	F	AAV8	4R uDys	8.90E + 13	CsA/MMF	2.77	24.33	21.57
Dog #9	GY	Affected	M	AAV8	4R uDys	1.09E + 14	Prednisolone	2.43	13.33	10.90
Dog #10	LY	Affected	M	AAV8	4R uDys	1.04E + 14	Prednisolone	2.50	13.70	11.20
Dog #11	LY	Affected	M	AAV9	AP	1.92E + 14	CsA/MMF	1.93	5.43	3.50
Dog #12	XY	Normal	M	AAV9	AP	9.06E + 14	None	0.17	22.17	22.00
Dog #13	GW	Affected	F	AAV9	5R uDys	1.00E + 14	CsA/MMF	3.60	11.47	7.87
Dog #14	GL	Affected	F	AAV9	5R uDys	5.00E + 13	CsA/MMF	3.53	43.17	39.63
Dog #15	WY	Affected	M	AAV9	5R uDys	1.00E + 14	CsA/MMF	3.63	43.27	39.63
Dog #16	LY	Affected	M	AAV9	5R uDys	5.05E + 14	Prednisolone	2.60	42.67	40.07
Dog #17	LY	Affected	M	AAV9	5R uDys	3.00E + 14	Prednisolone	2.67	42.73	40.07
Dog #18	XX	Normal	F	None	None	None	None	None	8.00	None
Dog #19	XY	Normal	M	None	None	None	None	None	6.77	None
Dog #20	XY	Normal	M	None	None	None	None	None	8.23	None
Dog #21	GY	Affected	M	None	None	None	None	None	7.30	None
Dog #22	GW	Affected	F	None	None	None	None	None	6.83	None
Dog #23	LW	Affected	F	None	None	None	None	None	7.13	None

Abbreviations: 4R uDys, four‐repeat microdystrophin; 5R uDys, five‐repeat microdystrophin; AP, alkaline phosphatase; CsA, cyclosporine; G, golden retriever muscular dystrophy mutation (point mutation in intron 6); kg, kilogram; L, Labrador retriever muscular dystrophy mutation (repetitive element insertion in intron 19); MMF, mycophenolate mofetil; vg, vector genome; W, Welsh corgi muscular dystrophy mutation (repetitive element insertion in intron 13); X, normal X chromosome; Y, normal Y chromosome.

### 2.2 AAV Vector and Systemic Delivery

The AP vector expressed heat‐resistant human placental AP from the ubiquitous RSV promoter. uDys was expressed from the muscle‐specific CK8e promoter. A four‐repeat uDys was packaged in AAV8. This uDys carries the N‐terminal domain, hinge 1, spectrin‐like repeats R1, R16, R17 and R24, hinge 4 and the cysteine‐rich domain [18]. A five‐repeat uDys was packaged in AAV9. This uDys has an additional spectrin‐like repeat (R23) [19]. The AAV9 uDys vector was manufactured at the University of Pennsylvania Vector Core. Other AAV vectors were manufactured in‐house using our published protocol [20]. AAV titre was determined by TaqMan qPCR (Table S1). The AAV dose, age at injection, age at termination and immune suppression regimen are presented in Table 1.

### 2.3 Morphometric Analysis

To correlate transgene expression with the fibre type/size of individual myofibres, we used immunofluorescence and histochemical staining. Muscles were embedded in Tissue Plus Optimal Cutting Temperature media (Scigen Scientific, Gardena, CA) and snap‐frozen in 2‐methylbutane cooled by liquid nitrogen. Ten‐micron cryosections were stained for AP activity or immunostained for uDys, laminin and myofibre types using published protocols [10, 19]. Antibodies used for immunostaining are shown in Table S2. Slides were visualized at the identical exposure setting using a Nikon E800 fluorescence microscope. Photomicrographs (five random 20× fields/slide) were captured with a Leica DFC7000T camera. Myofibre type and size were quantified using the MyoVision 1.0 software (https://www.uky.edu/chs/center‐for‐muscle‐biology/myovision). Fibre type and size quantification results were validated manually. AP and uDys expression were manually quantified and correlated to myofibre type results using the ImageJ (FIJI) cell‐counter plugin (https://imagej.net/software/fiji/). The analysis with the MyoVision 1.0 software was performed blinded, but manual quantification was not. Three different fibre type analyses were performed using different equations. We first quantified the overall fibre type composition in each muscle sample. Fibre type distribution (%) = (number of fibres of a particular fibre type)/(number of total fibres). We then quantified the fibre type composition of transgene‐positive myofibres. Transgene‐positive myofibre distribution (%) = (number of transgene‐positive fibres of a particular fibre type)/(number of all transgene‐positive fibres). Finally, we quantified the percentage of transgene‐positive myofibres in each fibre type. Fibre type normalized transgene‐positive myofibre distribution (%) = (number of transgene‐positive fibres of a particular fibre type)/(number of fibres of the same fibre type).

### 2.4 Statistics

Data were presented as mean ± SEM. Analyses were performed using Matlab Version R2023b Statistics and Machine Learning Toolbox Version 23.2 (MathWorks, Natick, MA). Data normality was determined using the Anderson–Darling test. Statistical significance between the two groups was determined using Student's *t*‐test for parametric data and the Wilcoxon rank sum test for non‐parametric data. Statistical comparisons between three or more groups were determined using one‐way ANOVA followed by the Tukey–Kramer multiple comparison test for parametric data and the Kruskal–Wallis test followed by Dunn's multiple comparison test for non‐parametric data. To determine whether AAV‐mediated expression had a fibre size preference in different types of myofibres, we analysed the data using a generalized linear regression model with a gamma distribution, followed by the Wilcoxon rank sum test. A *p* < 0.05 was considered statistically significant.

## 3 Results

### 3.1 Systemic AAV Delivery Resulted in Mosaic Transgene Expression in the Biceps Femoris, Teres Major and Latissimus Dorsi

**FIGURE 1:**
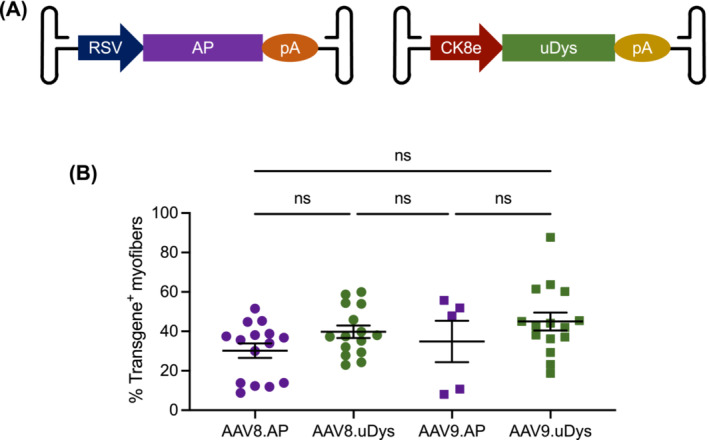
Quantification of transgene expression in muscles from systemic AAV‐injected dogs. (A) Cartoon illustration of AAV vectors used in the study. In the alkaline phosphatase (AP) vector, the heat‐resistant human placental AP gene was expressed from the ubiquitous RSV promoter. In the microdystrophin (uDys) vector, the uDys gene was expressed from the muscle‐specific CK8e promoter. (B) Quantification of transgene‐positive myofibres. Five dogs (two affected and three carrier) received systemic AAV8 AP vector injection. Two dogs (one affected and one normal) received systemic AAV9 AP vector injection. Five affected dogs received systemic AAV8 uDys vector injection. Five affected dogs received systemic AAV9 uDys vector injection. Two muscles (biceps femoris and latissimus dorsi) were collected from the AAV9 AP vector–injected affected dog. Three muscles (biceps femoris, teres major and latissimus dorsi) were collected from each dog for the remaining dogs. Each data point represents the result from one muscle in one dog. ns, not significant.

We packaged the AP and uDys expression cassettes in AAV8 and AAV9 and delivered intravenously to 17 dogs (Figures 1, S1 and S2 and Table 1). The AAV dose was 1.94 ± 0.52 × 10^14^ vg/kg (range 0.50–9.06 × 10^14^ vg/kg). The age at injection was 2.86 ± 0.30 months (range: 0.07–4.53 months). The age at harvest was 20.79 ± 3.30 months (range: 5.43–43.27 months). Transgene expression was examined in biceps femoris, teres major and latissimus dorsi. A moderate dose response was seen in the AP vector–injected dogs (Pearson *r* = 0.5269, *p* = 0.017) (Figure S1). Intriguingly, no statistically significant dose response was observed in the uDys vector–injected dogs (Pearson *r* = 0.2737, *p* = 0.143) (Figure S1). Visually, all three muscles displayed mosaic transgene expression irrespective of AAV serotype, transgene and dose (Figure S2). Hence, data from three muscles were combined. In dogs that received AAV8 and AAV9 AP vectors, the percentage of AP‐positive myofibres was 30.20% ± 3.67% (range: 8.75%–51.53%) and 34.86% ± 10.48% (range: 8.05%–55.71%), respectively (Figure 1B). In dogs that received AAV8 and AAV9 uDys vectors, the percentage of uDys‐positive myofibres was 39.80% ± 3.15% (range: 22.98%–59.94%) and 45.00% ± 4.42% (range: 18.69%–87.74%), respectively (Figure 1B). We did not detect statistically significant differences between AAV8 and AAV9 and between the AP and uDys vectors (Figure 1B).

### 3.2 AP Vectors Did Not Show a Fibre Type Preference in Dog Muscles

**FIGURE 2:**
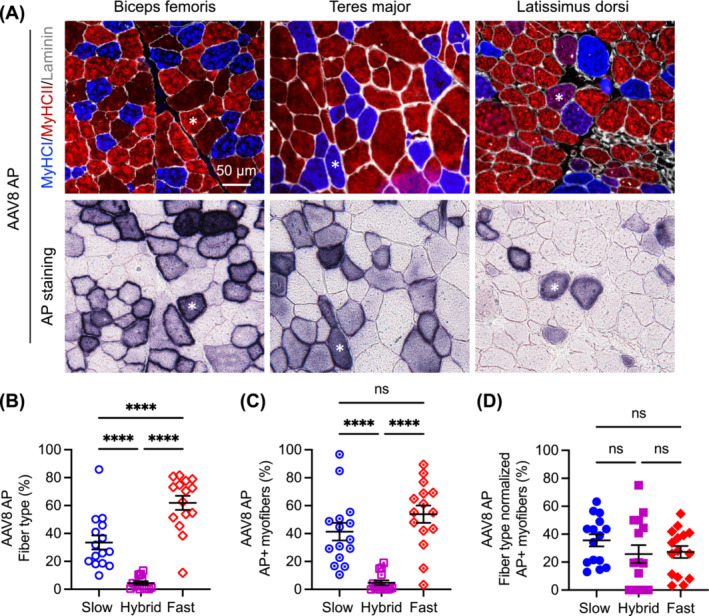
Evaluation of myofibre type composition and AP expression following systemic injection of the AAV8 AP reporter vector. Five dogs (two affected and three carrier dogs) received the vector. Three muscles (biceps femoris, teres major and latissimus dorsi) were examined in each dog. (A) Representative photomicrographs of myofibre type immunofluorescence staining and AP histochemical staining. Asterisk, the same myofibre in serial sections. MyHC, myosin heavy chain. Blue, slow Type I myofibre; red, fast Type IIa/IIx myofibre; purple, hybrid myofibre with both Type I and Type IIa/IIx fibres; green, Type IIb myofibre (note: no Type IIb fibre was detected); white, laminin. (B) Myofibre type composition. Each point represents data from one muscle in one dog. (C) Fibre type composition of AP‐positive myofibres. Each point represents data from one muscle in one dog. (D) Fibre type normalized AP expression. Each point represents data from one muscle in one dog. ns, not significant, ****p < 0.0001.

**FIGURE 3:**
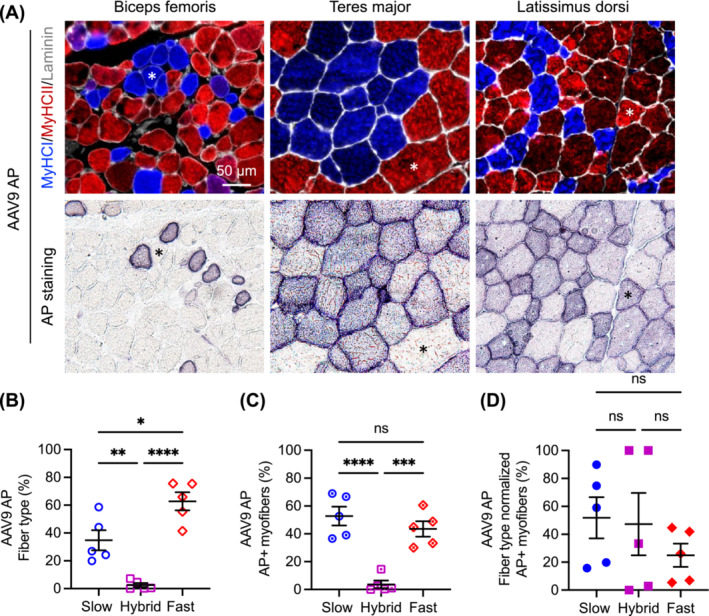
Evaluation of myofibre type composition and AP expression following systemic injection of the AAV9 AP reporter vector. Two dogs (one affected and one normal) received the vector. Three muscles (biceps femoris, teres major and latissimus dorsi) were examined in the normal dog. Two muscles (biceps femoris and latissimus dorsi) were examined in the affected dog. (A) Representative photomicrographs of myofibre type immunofluorescence staining and AP histochemical staining. Asterisk, the same myofibre in serial sections. MyHC, myosin heavy chain. Blue, slow Type I myofibre; red, fast Type IIa/IIx myofibre; purple, hybrid myofibre with both Type I and Tpe IIa/IIx fibres; green, Type IIb myofibre (note: no Type IIb fibre was detected); white, laminin. (B) Myofibre type composition. Each point represents the percentage of the corresponding myofibre type from one muscle. (C) Fibre type composition of AP‐positive myofibres. Each point represents data from one muscle in one dog. (D) Fibre type normalized AP expression. Each point represents data from one muscle in one dog. ns, not significant, *p < 0.05, **p < 0.01, ***p < 0.001, ****p < 0.0001.

In AP vector–injected dogs, we detected Type I, Type IIa/IIx and hybrid fibres, but not Type IIb fibres (Figures 2 and 3). In dogs that received the AAV8 AP vector, there were 33.56% ± 4.98% (range: 10.00%–85.93%) slow fibres, 61.94% ± 5.04% (range: 11.85%–81.70%) fast fibres and 4.50% ± 1.21% (range: 0.16%–13.37%) hybrid fibres (Figure 2B). AP‐positive cells comprised 41.36% ± 6.29% (range: 10.58%–96.77%) slow fibres, 53.88% ± 6.22% (range: 3.23%–89.42%) fast fibres and 4.77% ± 1.73% (range: 0.00%–19.05%) hybrid fibres (Figure 2C). After normalizing AP‐positive cells by the fibre type composition, AP expression was detected in 35.61% ± 4.35% (range: 12.93%–63.27%) of slow fibres, 27.27% ± 4.30% (range: 3.13%–54.57%) of fast fibres and 25.74% ± 6.43% (range: 0.00%–75.00%) of hybrid fibres (Figure 2D). There was no significant difference in AP expression among slow, fast and hybrid fibres (Figure 2D).

In dogs that received the AAV9 AP vector, there were 34.78% ± 7.25% (range: 19.90%–58.65%) slow fibres, 62.71% ± 6.51% (range: 41.35%–75.57%) fast fibres and 2.51% ± 1.46% (range: 0.00%–7.20%) hybrid fibres (Figure 3B). AP‐positive cells consisted of 52.79% ± 6.72% (range: 36.59%–68.93%) slow fibres, 43.50% ± 5.50% (range: 30.10%–60.57%) fast fibres and 3.71% ± 2.77% (range: 0.00%–14.63%) hybrid fibres (Figure 3C). After normalizing AP‐positive cells by the fibre type composition, AP expression was detected in 51.82% ± 14.75% (range: 15.75%–89.86%) of slow fibres, 24.99% ± 8.30% (range: 5.47%–44.65%) of fast fibres and 47.25% ± 22.31% (range: 0.00%–100.00%) of hybrid fibres (Figure 3D). There was no significant difference in AP expression among slow, fast and hybrid fibres (Figure 3D).

### 3.3 Fibre Type Distribution in Affected and Non‐affected Muscles

Both non‐affected (normal and carrier) and affected dogs received the AP vector (Figure S3). In non‐affected dogs, slow, fast and hybrid fibres constituted 32.83% ± 3.99% (range: 18.14%–58.65%), 66.06% ± 4.03% (range: 41.35%–81.70%) and 1.13% ± 0.41% (range: 0.00%–4.35%), respectively. In affected dogs, slow, fast and hybrid fibres constituted 35.42% ± 8.60% (range 10.00%–85.93%), 56.26% ± 7.99% (range: 11.85%–78.98%) and 8.32% ± 1.29% (range: 2.22%–13.37%), respectively. There were no significant differences in slow and fast fibre type distributions between non‐affected and affected dogs.

### 3.4 uDys Was Equally Expressed in Slow and Fast Fibres

**FIGURE 4:**
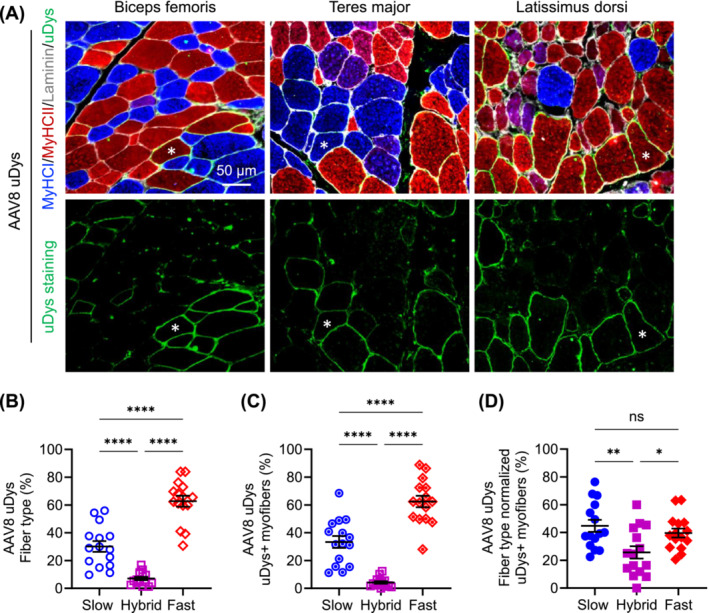
Evaluation of myofibre type composition and uDys expression following systemic injection of the AAV8 uDys vector. Five affected dogs received the vector. Three muscles (biceps femoris, teres major and latissimus dorsi) were examined in each dog. (A) Representative photomicrographs of myofibre type and uDys immunofluorescence staining. Asterisk, the same myofibre. MyHC, myosin heavy chain. Blue in the cytosol, slow Type I myofibre; red in the cytosol, fast Type IIa/IIx myofibre; purple in the cytosol, hybrid myofibre with both Type I and Type IIa/IIx fibres; green in the cytosol, Type IIb myofibre (note: no Type IIb fibre was detected); green on the sarcolemma, uDys; white on the sarcolemma, laminin. (B) Myofibre type composition. Each point represents data from one muscle in one dog. (C) Fibre type composition of uDys‐positive myofibres. Each point represents data from one muscle in one dog. (D) Fibre type normalized uDys expression. Each point represents data from one muscle in one dog. ns, not significant, *p < 0.05, **p < 0.01, ****p < 0.0001.

**FIGURE 5:**
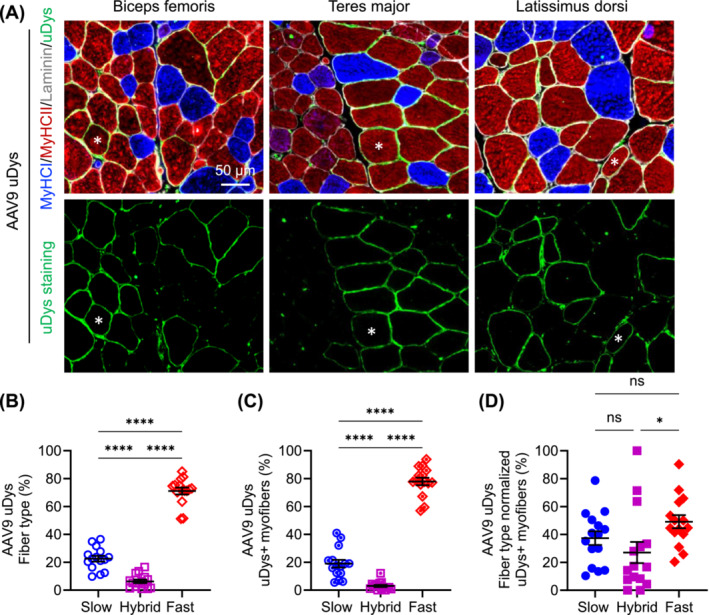
Evaluation of myofibre type composition and uDys expression following systemic injection of the AAV9 uDys vector. Five affected dogs received the vector. Three muscles (biceps femoris, teres major and latissimus dorsi) were examined in each dog. (A) Representative photomicrographs of myofibre type and uDys immunofluorescence staining. Asterisk, the same myofibre. MyHC, myosin heavy chain. Blue in the cytosol, slow Type I myofibre; red in the cytosol, fast Type IIa/IIx myofibre; purple in the cytosol, hybrid myofibre with both Type I and Type IIa/IIx fibres; green in the cytosol, Type IIb myofibre (note: no type IIb fibre was detected); green on the sarcolemma, uDys; white on the sarcolemma, laminin. (B) Myofibre type composition. Each point represents data from one muscle in one dog. (C) Fibre type composition of uDys‐positive myofibres. Each point represents data from one muscle in one dog. (D) Fibre type‐normalized uDys expression. Each point represents data from one muscle in one dog. ns, not significant, *p < 0.05, ****p < 0.0001.

Consistent with what we observed in AP vector–injected dogs, Type IIb fibres were not detected in uDys vector–injected dogs (Figures 4 and 5). In dogs that received the AAV8 uDys vector (Figure 4), there were 30.16% ± 3.87% (range: 9.74%–55.92%) slow fibres, 62.71% ± 4.00% (range: 30.70%–83.98%) fast fibres and 7.13% ± 1.22% (range 1.24%–16.89%) hybrid fibres (Figure 4B). uDys‐positive cells consisted of 33.37% ± 4.24% (range: 11.23%–68.49%) slow fibres, 62.42% ± 4.15% (range: 28.08%–88.77%) fast fibres and 4.21% ± 0.90% (range: 0.00%–12.35%) hybrid fibres (Figure 4C). After normalizing uDys‐positive cells by the fibre type composition, uDys expression was detected in 44.79% ± 4.31% (range: 22.45%–76.43%) of slow fibres, 39.61% ± 3.20% (range: 20.57%–63.36%) of fast fibres and 25.69% ± 4.47% (range: 0.00%–60.00%) of hybrid fibres (Figure 4D). uDys expression in slow and fast fibres was significantly higher than that in hybrid fibres. However, no significant difference was detected between slow and fast fibres (Figure 4D).

In dogs that received the AAV9 uDys vector (Figure 5), there were 22.60% ± 2.08% (range: 9.73%–36.50%) slow fibres, 71.10% ± 2.45% (range: 51.21%–85.04%) fast fibres and 6.28% ± 1.26% (range: 0.93%–16.58%) hybrid fibres (Figure 5B). uDys‐positive cells consisted of 19.02% ± 2.77% (range: 5.56%–40.91%) slow fibres, 77.97 ± 2.68% (range: 57.03%–93.87%) fast fibres and 3.02% ± 0.80% (range: 0.00–12.10%) hybrid fibres (Figure 5C). After normalizing uDys‐positive cells by the fibre type composition, uDys expression was detected in 37.38% ± 4.93% (range: 10.34%–78.67%) of slow fibres, 49.14% ± 4.69% (range: 20.42%–90.38%) of fast fibres and 27.04% ± 7.58% (range: 0.00%–100.00%) of hybrid fibres (Figure 5D). uDys expression in fast fibres was significantly higher than that in hybrid fibres. uDys expression in slow fibres was higher than that in hybrid fibres but did not reach statistical significance. No significant difference was detected between slow and fast fibres (Figure 5D).

### 3.5 uDys Expression Did Not Alter Fibre Type Composition

We compared the fibre type distribution between uDys‐positive and uDys‐negative fibres (Figure S4). In both groups of myofibres, the most prevalent fibre type was fast fibres, followed by slow fibres and then hybrid fibres. Except for hybrid fibres in the teres major, there were no significant differences between uDys‐positive and uDys‐negative fibres in all three fibre types.

### 3.6 AP Vector Preferred Smaller Fibres

**FIGURE 6:**
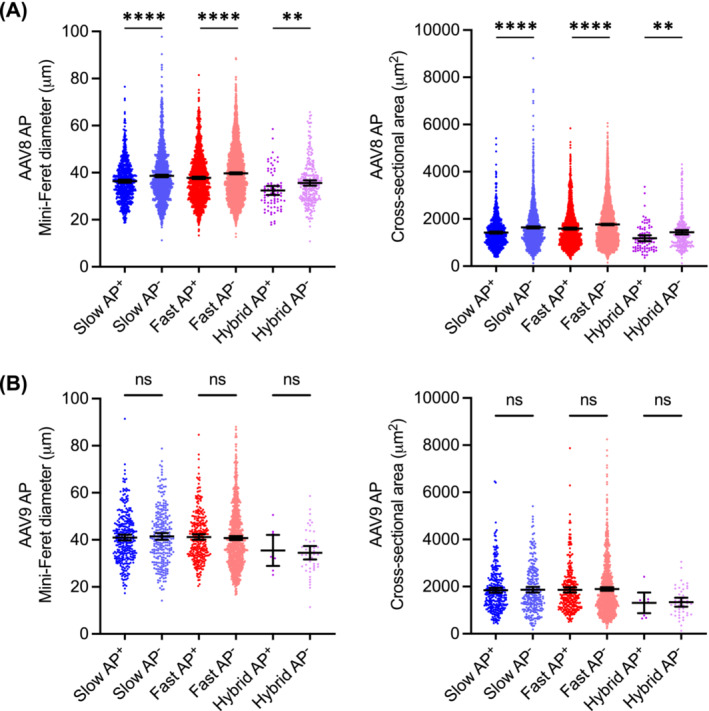
Evaluation of fibre size distribution in AP‐positive and AP‐negative myofibres following systemic injection of the AP reporter vector. Five dogs (two affected and three carrier dogs) received the AAV8 AP vector. Two dogs (one affected and one normal) received the AAV9 AP vector. Two muscles (biceps femoris and latissimus dorsi) were examined in the AAV9 AP vector–injected affected dog. Three muscles (biceps femoris, teres major and latissimus dorsi) were examined in the remaining dogs. (A) Scatter plots of the mini‐Feret diameter (left panel) and cross‐sectional area (right panel) of individual myofibres in dogs that received the AAV8 AP vector. AP‐positive slow fibres, *N* = 899; AP‐negative slow fibres, *N* = 1677; AP‐positive fast fibres, *N* = 1639; AP‐negative fast fibres, *N* = 3622; AP‐positive hybrid fibres, *N* = 80; AP‐negative hybrid fibres, *N* = 262. (B) Scatter plots of the mini‐Feret diameter (left panel) and cross‐sectional area (right panel) of individual myofibres in dogs that received the AAV9 AP vector. AP‐positive slow fibres, *N* = 314; AP‐negative slow fibres, *N* = 263; AP‐positive fast fibres, *N* = 301; AP‐negative fast fibres, *N* = 945; AP‐positive hybrid fibres, *N* = 9; AP‐negative hybrid fibres, *N* = 45. Data are presented as mean ± 95% confidence interval. Each point represents data from one myofibre. ns, not significant, **p < 0.01, ****p < 0.0001.

To investigate the fibre size preference, we quantified the mini‐Feret diameter and the cross‐sectional area in AP‐positive and AP‐negative myofibres (Figures 6 and S5–S7). In dogs that received the AAV8 AP vector (Figure 6A), the mini‐Feret diameter was 36.12 ± 0.28 μm (range: 18.75–76.52 μm) and 38.59 ± 0.26 μm (range: 11.25–97.76 μm) in AP‐positive and AP‐negative slow myofibres, respectively; was 37.58 ± 0.25 μm (range: 13.32–81.50 μm) and 39.76 ± 0.19 μm (range: 12.64–88.67 μm) in AP‐positive and AP‐negative fast myofibres, respectively; and was 32.39 ± 0.92 μm (range: 17.89–58.55 μm) and 35.63 ± 0.58 μm (range: 10.80–65.75 μm) in AP‐positive and AP‐negative hybrid myofibres, respectively. The cross‐sectional area was 1422.50 ± 20.90 μm^2^ (range 392.74–5414.53 μm^2^) and 1641.94 ± 22.27 μm^2^ (range 123.06–8812.10 μm^2^) in AP‐positive and negative slow myofibres, respectively; was 1590.46 ± 20.27 μm^2^ (range 297.12–5842.23 μm^2^) and 1766.06 ± 15.59 μm^2^ (range 151.76–6060.03 μm^2^) in AP‐positive and negative fast myofibres, respectively; was 1183.25 ± 64.15 μm^2^ (range 356.40–3361.85 μm^2^) and 1436.12 ± 46.43 μm^2^ (range 122.43–4314.38 μm^2^) in AP‐positive and negative hybrid myofibres, respectively. AP‐positive fibres were significantly smaller than AP‐negative fibres (Figure 6A).

Two affected dogs and three carrier dogs received the AAV8 AP vector. In affected dogs, the fibre size of AP‐positive myofibres was significantly smaller (Figure S6A). In carrier dogs, the fibre size of AP‐positive fast myofibres was significantly smaller. There was also a trend of smaller fibre size in AP‐positive slow and hybrid myofibres (Figure S6B).

One normal and one affected dog received the AAV9 AP vector (Figures 6B, S5 and S7). When data from these two dogs were combined, there was no significant fibre size difference between AP‐positive and AP‐negative fibres in all three fibre types (Figure 6B). However, when data from these two dogs were analysed separately (Figure S7), AP‐positive slow and fast fibres were significantly smaller in the normal dog (Figure S7B).

Collectively, AP expression was enriched in smaller fibres in six of seven dogs that received the AAV‐AP vector.

### 3.7 Systemic uDys Gene Therapy Prevented Myofibre Atrophy

**FIGURE 7:**
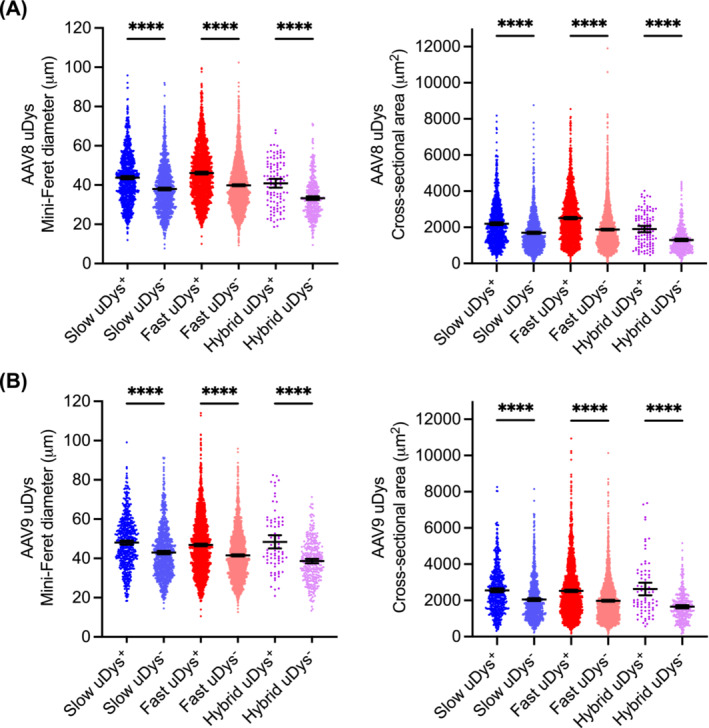
Evaluation of fibre size distribution in uDys‐positive and uDys‐negative myofibres following systemic injection of the uDys vector. Five affected dogs received the AAV8 uDys vector. Five affected dogs received the AAV9 uDys vector. Three muscles (biceps femoris, teres major and latissimus dorsi) were examined in each dog. (A) Scatter plots of the mini‐Feret diameter (left panel) and cross‐sectional area (right panel) of individual myofibres in dogs that received the AAV8 uDys vector. uDys‐positive slow fibres, *N* = 946; uDys‐negative slow fibres, *N* = 1261; uDys‐positive fast fibres, *N* = 1762; uDys‐negative fast fibres, *N* = 2870; uDys‐positive hybrid fibres, *N* = 109; uDys‐negative hybrid fibres, *N* = 444. (B) Scatter plots of the mini‐Feret diameter (left panel) and cross‐sectional area (right panel) of individual myofibres in dogs that received the AAV9 uDys vector. uDys‐positive slow fibres, *N* = 573; uDys‐negative slow fibres, *N* = 908; uDys‐positive fast fibres, *N* = 2205; uDys‐negative fast fibres, *N* = 2293; uDys‐positive hybrid fibres, *N* = 78; uDys‐negative hybrid fibres, *N* = 329. Data are presented as mean ± 95% confidence interval. Each point represents data from one myofibre. ****p < 0.0001.

In the absence of AAV injection, normal dogs had a significantly larger mini‐Feret diameter and cross‐sectional area than affected dogs in all three fibre types (Figure S8). In affected dogs that received the AAV8 uDys vector (Figures 7A and S9A,B), the mini‐Feret diameter was 43.78 ± 0.43 μm (range: 12.02–95.83 μm) and 37.96 ± 0.34 μm (range: 7.71–92.03 μm) in uDys‐positive and uDys‐negative slow myofibres, respectively; was 46.10 ± 0.34 μm (range: 10.04–99.57 μm) and 39.87 ± 0.23 μm (range: 9.26–102.39 μm) in uDys‐positive and uDys‐negative fast myofibres, respectively; and was 40.89 ± 1.12 μm (range: 18.63–67.95 μm) and 33.30 ± 0.47 μm (range: 9.41–71.20 μm) in uDys‐positive and uDys‐negative hybrid myofibres, respectively. The cross‐sectional area was 2199.36 ± 39.92 μm^2^ (range: 154.01–8175.11 μm^2^) and 1698.25 ± 29.48 μm^2^ (range: 112.41–8757.33 μm^2^) in uDys‐positive and uDys‐negative slow myofibres, respectively; was 2507.36 ± 32.75 μm^2^ (range: 108.15–8547.80 μm^2^) and 1876.10 ± 21.63 μm^2^ (range: 86.72–11904.52 μm^2^) in uDys‐positive and uDys‐negative fast myofibres, respectively; was 1903.73 ± 88.59 μm^2^ (range: 466.43–4018.63 μm^2^) and 1299.98 ± 34.60 μm^2^ (range: 111.78–4518.39 μm^2^) in uDys‐positive and uDys‐negative hybrid myofibres, respectively. uDys‐positive myofibres were significantly larger than uDys‐negative myofibres irrespective of the fibre type (Figure 7A). Specifically, the mini‐Feret diameter was 15%, 16% and 23% larger in uDys‐positive slow, fast and hybrid fibres, respectively. The cross‐sectional area was 30%, 34% and 46% larger in uDys‐positive slow, fast and hybrid fibres, respectively.

In affected dogs that received the AAV9 uDys vector (Figures 7B and S9C,D), the mini‐Feret diameter was 48.03 ± 0.54 μm (range: 18.36–99.12 μm) and 43.00 ± 0.43 μm (range: 14.51–91.24 μm) in uDys‐positive and uDys‐negative slow myofibres, respectively; was 46.85 ± 0.31 μm (range: 10.51–114.07 μm) and 41.52 ± 0.26 μm (range: 12.65–95.88 μm) in uDys‐positive and uDys‐negative fast myofibres, respectively; and was 48.42 ± 1.68 μm (range 20.84–82.48 μm) and 38.63 ± 0.58 μm (range: 13.30–71.28 μm) in uDys‐positive and uDys‐negative hybrid myofibres, respectively. The cross‐sectional area was 2557.20 ± 52.71 μm^2^ (range: 314.79–8265.84 μm^2^) and 2046.16 ± 39.70 μm^2^ (range: 224.57–8148.55 μm^2^) in uDys‐positive and uDys‐negative slow myofibres, respectively; was 2528.75 ± 31.43 μm^2^ (range: 166.17–10938.21 μm^2^) and 1981.63 ± 24.03 μm^2^ (range: 142.11–10132.80 μm^2^) in uDys‐positive and uDys‐negative fast myofibres, respectively; and was 2630.37 ± 174.21 μm^2^ (range: 572.82–7367.95 μm^2^) and 1653.61 ± 44.56 μm^2^ (range: 162.54–5162.77 μm^2^) in uDys‐positive and uDys‐negative hybrid myofibres, respectively. uDys‐positive myofibres were significantly larger than uDys‐negative myofibres in all three fibre types (Figure 7B). Specifically, the mini‐Feret diameter was 12%, 13% and 25% larger in uDys‐positive slow, fast and hybrid fibres, respectively. The cross‐sectional area was 25%, 28% and 59% larger in uDys‐positive slow, fast and hybrid fibres, respectively.

In the AAV9 uDys study, four doses (0.5–5 × 10^14^ vg/kg) were used in five affected dogs. We analysed the size of uDys‐positive and uDys‐negative fibres according to the vector dose (Figure S10). In the dog that received the lowest dose (5 × 10^13^ vg/kg), the size of uDys‐positive slow fibres was significantly larger than that of uDys‐negative slow fibres (Figure S10A). In dogs that received 1–3 × 10^14^ vg/kg, uDys‐positive slow and fast fibres were significantly larger than the corresponding uDys‐negative fibres (Figure S10B,C). In the dog that received the highest dose (5 × 10^14^ vg/kg), the size of uDys‐positive fibres was larger, and it reached statistical significance for fast and hybrid fibres (Figure S10D).

## 4 Discussion

In this study, we evaluated the fibre type and fibre size tropism of systemically delivered AAV8 and AAV9 in dog muscles. We selected three muscles that showed mosaic transgene expression. Consistent with the literature, these muscles did not contain Type IIb myofibres, a characteristic feature of human and dog, but not mouse muscles [13]. Fibre type profiling revealed equally efficient transduction of slow and fast fibres by AAV8 and AAV9. Fibre size analysis suggests that the AP vector preferred smaller fibres. However, the size of uDys‐positive fibres was significantly larger than uDys‐negative fibres in affected dogs that received AAV uDys therapy.

Effective gene therapy hinges on efficient delivery of the therapeutic vector to the target cells. In muscle, the target cells consist of myofibres of different fibre types. Many studies have shown that AAV serotypes display distinctive fibre type preferences. Louboutin et al. found that AAV1 preferred fast fibres, whereas AAV5 preferred slow fibres [7]. Several studies suggest that AAV2 predominantly transduces slow fibres [4, 5, 6, 7]. We and others have shown that AAV9 more efficiently targeted fast fibres following systemic delivery [9, 10, 11], whereas Riaz et al. found that AAV9‐mediated expression was enriched in slow fibres following intramuscular injection [12]. It is worth noting that the fibre type tropism of AAV serotypes is also influenced by muscle regeneration and disease. For example, AAV1 was less efficient in transducing regenerated myofibres, whereas AAV2 showed enhanced transduction of regenerating myofibres [21, 22]. In normal mouse muscle, AAV6 transduced slow and fast fibres at similar efficiency [6]. However, in the mdx mouse model of DMD, AAV6 showed a trend towards preferential transduction of fast fibres [8]. Collectively, these studies suggest that fibre type is an important determining factor of AAV tropism in muscle. Although these published studies suggest that not all fibre types are equally permissive to a particular AAV serotype, these findings are of limited translational significance because they were all performed in rodent muscles that have abundant Type IIb fibres [13].

To better understand the fibre type tropism of AAV in large mammals, we retrospectively analysed fibre type and fibre size preference in 17 dogs that had received systemic AAV8 or AAV9 injections. AAV8 has been used in uDys gene therapy trials to treat DMD by Genethon and Regenxbio, whereas AAV9 has been used in uDys trials by Pfizer and Solid Biosciences [23].

Because the fibre type distribution exhibits a mosaic pattern, we initially hypothesized that mosaic transgene expression might correlate to the fibre type distribution. In other words, the preferential transduction of a particular fibre type might have resulted in mosaic transgene expression. We first examined the fibre type composition of three target muscles. Consistent with the literature [14], fast fibres were the most abundant type (62%–71%), followed by slow fibres (22%–35%). Hybrid fibres only constituted a small proportion (3%–7%) (Figures 2B, 3B, 4B, and 5B).

We observed a similar trend of fibre type distribution of transgene‐positive cells in AAV8 and AAV9 injected dogs (Figures 2C, 3C, 4C, and 5C), suggesting these two AAV serotypes have similar fibre type preferences. When transgene‐positive cells were normalized by the fibre type distribution, we did not detect a significant difference among all three fibre types in AP vector–injected dogs, suggesting AAV8 and AAV9 transduce slow, fast and hybrid fibres at similar efficiency. Intriguingly, in uDys vector–injected dogs, there was a reduction of uDys‐positive hybrid fibres (Figures 4D and 5D), which reached statistical significance for AAV8 (slow vs. hybrid and fast vs. hybrid) and AAV9 (fast vs. hybrid only). Hybrid fibres are transitional fibres [2, 3]. It is possible that uDys expression may have promoted the transition process, thereby resulting in fewer uDys‐positive hybrid fibres. Nonetheless, data from both AP and uDys vectors suggest that AAV8 and AAV9 are equally efficient in transducing slow and fast fibres, a finding consistent with our earlier AAV9 study in neonatal dogs [24]. Our results also align with the study of Muraine et al., in which the authors showed that AAV8 and AAV9 transduced slow and fast human muscle fibres at similar efficiency in a xenograft model [25]. In summary, our data suggest that the observed mosaic expression pattern is not due to mosaic fibre type distribution.

Fibre type tropism of AAV8 has never been investigated. However, several studies have examined the fibre type preference of AAV9 in mouse muscles [9, 10, 11, 12]. Interestingly, these studies revealed fibre type selective transduction. The differences between mouse results and dog results highlight the importance of selecting appropriate species when addressing translational questions. It should be pointed out that species‐dependent AAV serotype performance is not unique to skeletal muscle. For example, both AAV8 and AAV9 are highly efficient in transducing the mouse heart [9, 26]. However, only AAV8 works well for the dog heart [27].

Fibre size variation is a characteristic feature of muscle cells. However, only one study has examined the fibre size preference of AAV transduction [12]. The authors found that AAV6 transduction is primarily determined by the myofibre cross‐sectional area. To precisely determine the fibre size preference of AAV8 and AAV9, we quantified the mini‐Feret diameter and cross‐sectional area in both transgene‐positive and transgene‐negative cells (Figures 6, 7, S5–S7 and S9–S10). In dogs that received the AP vector, transgene expression was enriched in smaller fibres (Figures 6 and S5–S7). This suggests that the AP vector may preferentially transduce smaller‐size muscle cells. Alternatively, AP expression may have induced myofibre atrophy or prevented growth‐associated fibre size enlargement.

An important aspect of our study is determining whether uDys gene therapy improved fibre type composition and/or prevented fibre size atrophy in affected DMD dogs. A fast‐to‐slow fibre type transition has been reported in mdx mice and DMD patients [28, 29, 30]. It is believed that fast fibres are more susceptible to contraction‐induced damage because of their higher contraction speed and force production. Although a similar fast‐to‐slow transition was found in the diaphragm of affected dogs [31], recent studies revealed an unexpected slow‐to‐fast transition in the limb muscle (extensor carpi ulnaris and cranial tibialis) of DMD dogs [32, 33]. We examined the fibre type distribution in dogs that received the AP reporter vectors (Figure S3). In the three muscles, we studied, normal and carrier dogs were mainly composed of fast fibres. Surprisingly, the most prevalent fibre type was also fast fibres in affected dogs. There was no significant difference between affected and non‐affected dogs. Our results suggest some dystrophic muscles may lack fibre type transition. Analysis of the fibre type composition in uDys‐treated dogs provides further support to this notion. Specifically, we detected an identical fibre type distribution pattern in uDys‐positive and uDys‐negative fibres (Figure S4).

Quantitative studies suggest that the prevalent loss of large‐size fibres resulted in muscle atrophy in dystrophic muscles [34, 35]. Large‐size myofibres contain more contractile proteins and hence generate stronger force. Strategies aimed at increasing myofibre size (such as myostatin blockade) are being actively pursued to treat DMD [36]. We have previously shown that uDys therapy resulted in a shift toward larger myofibre size in severely affected D2‐mdx mice [19]. However, it is unclear whether uDys therapy can prevent dystrophic myofibre atrophy in large mammals.

We compared the myofibre size between normal and affected naïve dogs that did not receive AAV injection and between uDys‐positive and uDys‐negative fibres in AAV uDys‐treated affected dogs (Figures 7 and S8–S10). As expected, the fibre size of affected dogs was significantly smaller than that of normal dogs (Figure S8). In AAV uDys‐treated affected dogs, uDys‐expressing myofibres were significantly larger than myofibres that did not have uDys (Figures 7, S9, and S10). In contrast, in AP reporter vector–injected dogs, AP expression did not increase the fibre size (Figures 6 and S5–S7). Our results suggest that uDys therapy can prevent myofibre atrophy in a dystrophic large mammal.

Recently, Birch et al. examined the fibre diameter in the biceps femoris of uDys‐treated DMD dogs [37]. In dogs that received 1–2 × 10^14^ vg/kg uDys vectors, there were more fibres with larger diameters. However, the difference did not reach statistical significance. In our study, we quantified the fibre size of uDys‐positive and uDys‐negative fibres separately. This allows us to more precisely evaluate the impact of uDys expression on the fibre size of individual myofibres in the same muscle. In the Birch et al. study, the authors quantified the fibre size of all myofibres irrespective of uDys expression. As a significant proportion of myofibres was not transduced by the AAV‐uDys vector, the protective effect of uDys would be diluted due to the inclusion of uDys‐negative fibres in the Birch et al. study. In addition, Birch et al. collected tissues at 3 months post‐AAV injection while we collected tissues at 23.2 ± 4.7 months (range: 7.87–40.07) post‐AAV injection. It may take a longer time to visualize uDys‐mediated protection.

Our study has several limitations. First, dogs were injected and harvested at different ages. This may influence AAV transduction profiles. Second, different promoters were used to drive AP and uDys expression. We cannot rule out the possibility that promoter activity could differ between fibre types. A side‐by‐side comparison using the same promoter would further strengthen the conclusion. Third, the sample size is small, especially for the AAV9 AP group (only one affected and one normal dog received the vector). Future studies are needed to corroborate the findings. Fourth, in this study, we evaluated the fibre type and fibre size preference of AAV8 and AAV9. However, we did not study the myonuclei density. It is known that slow fibres have a higher myonuclei density than fast fibres [38, 39, 40]. Our results showed that slow and fast fibres were similarly transduced in dogs that received either the AP or the uDys vector (Figures 2, 3, 4, 5). This suggests that myonuclei density may not be a major factor in determining AAV transduction efficiency in different fibre types.

In summary, we comprehensively examined the fibre type and fibre size tropism of AAV8 and AAV9 in non‐affected and affected dogs using reporter and uDys vectors. We conclude that these two AAV serotypes are equally efficient in transducing slow and fast fibres in dog muscle. Importantly, we showed that uDys gene therapy significantly prevented dystrophic myofibre atrophy in the canine DMD model. Our findings provide important translational insight into AAV gene therapy for neuromuscular diseases.

## Author Contributions

D.D. conceived the idea and designed the study. M.J.B., B.M.B., J.A.T., D.O.P.L., Y.Y., X.P. and C.H. conducted experiments. M.J.B., G.Y., R.W.H. and D.D. analysed the data. M.J.B. and D.D. wrote the paper. M.J.B., B.M.B., G.Y., R.W.H. and D.D. edited the paper. All authors read the paper and approved the submission.

## Ethics Statement

All authors have certified that they comply with the ethical guidelines for authorship and publishing in the *Journal of Cachexia, Sarcopenia and Muscle*.

## Conflicts of Interest

D.D. is a member of the scientific advisory board for Solid Biosciences and an equity holder of Solid Biosciences. D.D. is a member of the scientific advisory board for Sardocor Corp. D.D. is an inventor of several issued and filed patents on AAV vector and DMD gene therapy. The Duan lab has received research support unrelated to this project from Elenae Therapeutics and Satellos Bioscience in the last 3 years. R.W.H. is serving on scientific advisory boards for Regeneron Pharmaceuticals–Intellia Therapeutics collaboration, Prevail Therapeutics, Pfizer and Biomarin and is also receiving funding from Roche. The other authors declare no conflicts of interest.

## Supporting information


**Figure S1** Dose response. (A) Correlation between the AAV dose and AP expression in seven dogs (Dogs #1–5, 11 and 12 in Table 1) that received the AAV‐AP vector. Three muscles (biceps femoris, teres major and latissimus dorsi) were examined in each injected dog except for one dog (Dog #11) in which the teres major was not examined. (B) Correlation between the AAV dose and uDys expression in ten dogs (Dogs #6–10 and 13–17 in Table 1) that received the AAV‐uDys vector. Three muscles (biceps femoris, teres major and latissimus dorsi) were examined in each injected dog. (C) Correlation between the AAV dose and transgene expression in all 17 AAV injected dogs. Each point represents one muscle from one dog. Best fit lines are calculated by simple linear regression.
**Figure S2.** Biceps femoris, teres major and latissimus dorsi showed mosaic transgene expression following systemic AAV injection. Representative photomicrographs of transgene expression in all 17 experimental dogs. (A) AAV8 AP vector–injected dogs (Dogs #1–5). (B) AAV9 AP vector–injected dogs (Dogs #11 and 12). (C) AAV8 uDys vector–injected dogs (Dogs #6–10). (D) AAV9 uDys vector–injected dogs (Dogs #13–17).
**Figure S3.** AAV‐AP–injected affected and non‐affected dogs showed similar fibre type distributions. (A) Overall fibre type composition of the biceps femoris, teres major and latissimus dorsi in four non‐affected dogs (one normal and three carriers; Dogs #3–5 and 12 in Table 1). (B) Overall fibre type composition of the biceps femoris, teres major and latissimus dorsi in three affected DMD dogs (Dogs #1, 2 and 11 in Table 1). Please note the teres major was not examined in Dog #11. (C) Comparison of overall fibre type distribution in affected and non‐affected dogs. (D) Comparison of fibre type distribution in the biceps femoris of affected and non‐affected dogs. (E) Comparison of fibre type distribution in the teres major of affected and non‐affected dogs. (F) Comparison of fibre type distribution in the latissimus dorsi of affected and non‐affected dogs. ns, not significant, *p < 0.05, **p < 0.01, ***p < 0.001, ****p < 0.0001.
**Figure S4.** Fibre type distributions of uDys‐positive and uDys‐negative fibres were similar in ten affected dogs that received the AAV uDys vector. (A) Comparison of fibre type distribution of uDys‐positive and uDys‐negative fibres in the biceps femoris. (B) Comparison of fibre type distribution of uDys‐positive and uDys‐negative fibres in the teres major. (C) Comparison of fibre type distribution of uDys‐positive and uDys‐negative fibres in the latissimus dorsi. ns, not significant, **p < 0.01.
**Figure S5.** Fibre size distributions in slow, fast and hybrid fibres of the AP vector–injected muscles. (A) Mini‐Feret diameter of AP‐positive and AP‐negative myofibres in slow, fast and hybrid myofibres in AAV8 AP vector–injected dogs. (B) Cross‐sectional area of AP‐positive and AP‐negative myofibres in slow, fast and hybrid myofibres in AAV8 AP vector–injected dogs. (C) Mini‐Feret diameter of AP‐positive and AP‐negative myofibres in slow, fast and hybrid myofibres in AAV9 AP vector–injected dogs. (D) Cross‐sectional area of AP‐positive and AP‐negative myofibres in slow, fast and hybrid myofibres in AAV9 AP vector–injected dogs. AAV8 AP‐positive slow fibres, *N* = 899; AAV8 AP‐negative slow fibres, *N* = 1677; AAV8 AP‐positive fast fibres, *N* = 1639; AAV8 AP‐negative fast fibres, *N* = 3622; AAV8 AP‐positive hybrid fibres, *N* = 80; AAV8 AP‐negative hybrid fibres, *N* = 262. AAV9 AP‐positive slow fibres, *N* = 314; AAV9 AP‐negative slow fibres, *N* = 263; AAV9 AP‐positive fast fibres, *N* = 301; AAV9 AP‐negative fast fibres, *N* = 945; AAV9 AP‐positive hybrid fibres, *N* = 9; AAV9 AP‐negative hybrid fibres, *N* = 45.
**Figure S6.** Evaluation of fibre size distribution in AP‐positive and AP‐negative myofibres following systemic injection of the AAV8 AP vector. (A) Scatter plots and histogram graphs of the mini‐Feret diameter (left panel) and cross‐sectional area (right panel) of AP‐positive and AP‐negative slow, fast and hybrid myofibres in AAV8 AP vector–injected affected dogs. In scatter plots, data are presented as mean ± 95% confidence interval, and each point represents data from one myofibre. AP‐positive slow fibres, *N* = 176; AP‐negative slow fibres, *N* = 797; AP‐positive fast fibres, *N* = 198; AP‐negative fast fibres, *N* = 1324; AP‐positive hybrid fibres, *N* = 55; AP‐negative hybrid fibres, *N* = 214. B, Scatter plots and histogram graphs of the mini‐Feret diameter (left panel) and cross‐sectional area (right panel) of AP‐positive and AP‐negative slow, fast, and hybrid myofibres in AAV8 AP vector–injected carrier dogs. In scatter plots, data are presented as mean ± 95% confidence interval, and each point represents data from one myofibre. AP‐positive slow fibres, *N* = 723; AP‐negative slow fibres, *N* = 880; AP‐positive fast fibres, *N* = 1441; AP‐negative fast fibres, *N* = 2298; AP‐positive hybrid fibres, *N* = 25; AP‐negative hybrid fibres, *N* = 48.
**Figure S7.** Evaluation of fibre size distribution in AP‐positive and AP‐negative myofibres following systemic injection of the AAV9 AP vector. (A) Scatter plots and histogram graphs of the mini‐Feret diameter (left panel) and cross‐sectional area (right panel) of AP‐positive and AP‐negative slow, fast and hybrid myofibres in the AAV9 AP vector–injected affected dog. In scatter plots, data are presented as mean ± 95% confidence interval, and each point represents data from one myofibre. AP‐positive slow fibres, *N* = 35; AP‐negative slow fibres, *N* = 168; AP‐positive fast fibres, *N* = 37; AP‐negative fast fibres, *N* = 562; AP‐positive hybrid fibres, *N* = 7; AP‐negative hybrid fibres, *N* = 45. B, Scatter plots and histogram graphs of the mini‐Feret diameter (left panel) and cross‐sectional area (right panel) of AP‐positive and AP‐negative slow, fast and hybrid myofibres in the AAV9 AP vector–injected normal dog. In scatter plots, data are presented as mean ± 95% confidence interval, and each point represents data from one myofibre. AP‐positive slow fibres, *N* = 276; AP‐negative slow fibres, *N* = 95; AP‐positive fast fibres, *N* = 264; AP‐negative fast fibres, *N* = 383; AP‐positive hybrid fibres, *N* = 2; AP‐negative hybrid fibres, *N* = 0.
**Figure S8.** Evaluation of fibre size distribution in slow, fast and hybrid myofibres of normal and affected dogs. Scatter plots and histogram graphs of the mini‐Feret diameter (left panel) and cross‐sectional area (right panel) in slow, fast and hybrid myofibres of three normal (WT) and three affected (DMD) dogs (Dogs #18–23 in Table 1). In scatter plots, data are presented as mean ± 95% confidence interval, and each point represents data from one myofibre. Slow fibres of affected dogs, *N* = 1903; slow fibres of normal dogs, *N* = 1819; fast fibres of affected dogs, *N* = 2421; fast fibres of normal dogs, *N* = 2665; hybrid fibres of affected dogs, *N* = 703; hybrid fibres of normal dogs, *N* = 395.
**Figure S9.** Fibre size distributions in slow, fast and hybrid fibres of the uDys vector–injected muscles. (A) Mini‐Feret diameter of uDys‐positive and uDys‐negative slow, fast and hybrid myofibres in AAV8 uDys vector–injected dogs. (B) Cross‐sectional area of uDys‐positive and uDys‐negative slow, fast and hybrid myofibres in AAV8 uDys vector–injected dogs. (C) Mini‐Feret diameter of uDys‐positive and uDys‐negative slow, fast and hybrid myofibres in AAV9 uDys vector–injected dogs. (D) Cross‐sectional area of uDys‐positive and uDys‐negative slow, fast, and hybrid myofibres in AAV9 uDys vector–injected dogs. AAV8 uDys‐positive slow fibres, *N* = 946; AAV8 uDys‐negative slow fibres, *N* = 1261; AAV8 uDys‐positive fast fibres, *N* = 1762; AAV8 uDys‐negative fast fibres, *N* = 2870; AAV8 uDys‐positive hybrid fibres, *N* = 109; AAV8 uDys‐negative hybrid fibres, *N* = 444. AAV9 uDys‐positive slow fibres, *N* = 573; AAV9 uDys‐negative slow fibres, *N* = 908; AAV9 uDys‐positive fast fibres, *N* = 2205; AAV9 uDys‐negative fast fibres, *N* = 2293; AAV9 uDys‐positive hybrid fibres, *N* = 78; AAV9 uDys‐negative hybrid fibres, *N* = 329.
**Figure S10.** Evaluation of fibre size distribution in uDys‐positive and uDys‐negative myofibres following systemic injection of the AAV9 uDys vector. (A) Scatter plots and histogram graphs of the mini‐Feret diameter (left panel) and cross‐sectional area (right panel) of uDys‐positive and uDys‐negative slow, fast and hybrid myofibres in an affected dog that received 5 × 10^13^ vg/kg of the AAV9 uDys vector. In scatter plots, data are presented as mean ± 95% confidence interval, and each point represents data from one myofibre. uDys‐positive slow fibres, *N* = 96; uDys‐negative slow fibres, *N* = 245; uDys‐positive fast fibres, *N* = 398; uDys‐negative fast fibres, *N* = 573; uDys‐positive hybrid fibres, *N* = 1; uDys‐negative hybrid fibres, *N* = 37. (B) Scatter plots and histogram graphs of the mini‐Feret diameter (left panel) and cross‐sectional area (right panel) of uDys‐positive and uDys‐negative slow, fast and hybrid myofibres in two affected dogs that received 1 × 10^14^ vg/kg of the AAV9 uDys vector. In scatter plots, data are presented as mean ± 95% confidence interval, and each point represents data from one myofibre. uDys‐positive slow fibres, *N* = 296; uDys‐negative slow fibres, *N* = 430; uDys‐positive fast fibres, *N* = 950; uDys‐negative fast fibres, *N* = 902; uDys‐positive hybrid fibres, *N* = 41; uDys‐negative hybrid fibres, *N* = 191. (C) Scatter plots and histogram graphs of the mini‐Feret diameter (left panel) and cross‐sectional area (right panel) of uDys‐positive and uDys‐negative slow, fast and hybrid myofibres in an affected dog that received 3 × 10^14^ vg/kg of the AAV9 uDys vector. In scatter plots, data are presented as mean ± 95% confidence interval, and each point represents data from one myofibre. uDys‐positive slow fibres, *N* = 79; uDys‐negative slow fibres, *N* = 139; uDys‐positive fast fibres, *N* = 417; uDys‐negative fast fibres, *N* = 492; uDys‐positive hybrid fibres, *N* = 7; uDys‐negative hybrid fibres, *N* = 41. (D) Scatter plots and histogram graphs of the mini‐Feret diameter (left panel) and cross‐sectional area (right panel) of uDys‐positive and uDys‐negative slow, fast and hybrid myofibres in an affected dog that received 5 × 10^14^ vg/kg of the AAV9 uDys vector. In scatter plots, data are presented as mean ± 95% confidence interval, and each point represents data from one myofibre. uDys‐positive slow fibres, *N* = 102; uDys12 negative slow fibres, *N* = 94; uDys‐positive fast fibres, *N* = 440; uDys‐negative fast fibres, *N* = 326; uDys‐positive hybrid fibres, *N* = 29; uDys‐negative hybrid fibres, *N* = 60.
