# Supplementary material for: Adeno‐Associated Virus 8 and 9 Myofibre Type/Size Tropism Profiling Reveals Therapeutic Effect of Microdystrophin in Canines
Source: J Cachexia Sarcopenia Muscle. 2025 Jan 10;16(1):e13681. doi: 10.1002/jcsm.13681 (PMC11718217; doi:10.1002/jcsm.13681)
Supplement: Supplementary file 1 — Figure S1 Dose response. (A) Correlation between the AAV dose and AP expression in seven dogs (Dogs #1–5, 11 and 12 in Table 1) that received the AAV‐AP vector. Three muscles (biceps femoris, teres major and latissimus dorsi) were examined in each injected dog except for one dog (Dog #11) in which the teres major was not examined. (B) Correlation between the AAV dose and uDys expression in ten dogs (Dogs #6–10 and 13–17 in Table 1) that received the AAV‐uDys vector. Three muscles (biceps femoris, teres major and latissimus dorsi) were examined in each injected dog. (C) Correlation between the AAV dose and transgene expression in all 17 AAV injected dogs. Each point represents one muscle from one dog. Best fit lines are calculated by simple linear regression. Figure S2. Biceps femoris, teres major and latissimus dorsi showed mosaic transgene expression following systemic AAV injection. Representative photomicrographs of transgene expression in all 17 experimental dogs. (A) AAV8 AP vector–injected dogs (Dogs #1–5). (B) AAV9 AP vector–injected dogs (Dogs #11 and 12). (C) AAV8 uDys vector–injected dogs (Dogs #6–10). (D) AAV9 uDys vector–injected dogs (Dogs #13–17). Figure S3. AAV‐AP–injected affected and non‐affected dogs showed similar fibre type distributions. (A) Overall fibre type composition of the biceps femoris, teres major and latissimus dorsi in four non‐affected dogs (one normal and three carriers; Dogs #3–5 and 12 in Table 1). (B) Overall fibre type composition of the biceps femoris, teres major and latissimus dorsi in three affected DMD dogs (Dogs #1, 2 and 11 in Table 1). Please note the teres major was not examined in Dog #11. (C) Comparison of overall fibre type distribution in affected and non‐affected dogs. (D) Comparison of fibre type distribution in the biceps femoris of affected and non‐affected dogs. (E) Comparison of fibre type distribution in the teres major of affected and non‐affected dogs. (F) Comparison of fibre type distribution in the latiss [file JCSM-16-e13681-s001.pdf]

Supporting Information

Adeno-associated virus-8/9 myofibre type/size tropism profiling reveals therapeutic effect of microdystrophin in canines

Matthew J. Burke[1], Braiden M. Blatt[1,2], James A. Teixeira[1], Dennis O. Pérez-López[1], Yongping Yue[1], Xiufang Pan[1], Chady H. Hakim[1], Gang Yao[3], Roland W. Herzog[4], Dongsheng Duan[1,3,5,6,*]

[1]Department of Molecular Microbiology and Immunology, School of Medicine, University of Missouri, Columbia, MO

[2]College of Veterinary Medicine, University of Missouri, Columbia, MO

[3]Department of Chemical and Biomedical Engineering, College of Engineering, University of Missouri, Columbia, MO

[4]Department of Pediatrics, Herman B Wells Center for Pediatric Research, Indiana University, Indianapolis, IN

[5]Department of Biomedical Sciences, College of Veterinary Medicine, University of Missouri, Columbia, MO

[6]Department of Neurology, School of Medicine, University of Missouri, Columbia, MO

*, Corresponding Address:

Dongsheng Duan PhD
Dep. of Molecular Microbiology and Immunology
One Hospital Dr.
Columbia, MO 65212
Phone: 573-884-9584
Fax: 573-882-4287
Email: duand@missouri.edu

Table S1. Primers and probes used for AAV titer determination

AAV AP	Forward primer	CACTCCCACGTCTTCTCCTT
	Reverse primer	TCCGTATAGGAGGACCGTGTAG
	Probe	6FAM-CCAGCCCGAAGATGGA-NFQ-MGB
AAV8 4-repeat uDys	Forward primer	GTGGCTAGCATGGAGAAGCA
	Reverse primer	CTCACATGGGTAATCTCAGTCAGA
	Probe	6FAM- CTGCACAGAGAAATCT-NFQ-MGB
AAV9 5-repeat uDys	Forward primer	GAGTCGCCTCTATGGAAAAGCA
	Reverse primer	GGTCAGATAAGTACTTGGCACGTAA
	Probe	6FAM-ATCTCTTTGTGCAGATTAC-NFQ-MGB

Table S2. Antibodies used in immunostaining

Primary antibody	Name	Antibody specifics	Supplier
Type I myosin heavy chain	BA-D5	Mouse monoclonal antibody, IgG2b (1:20 dilution)	Developmental Studies Hybridoma Bank, Iowa City, IA
Type IIa and IIx myosin heavy chain	SC-71	Mouse monoclonal antibody, IgG1 (1:100 dilution)	Developmental Studies Hybridoma Bank, Iowa City, IA
Type IIb myosin heavy chain	BF-F3	Mouse monoclonal antibody, IgM (1:50 dilution)	Developmental Studies Hybridoma Bank, Iowa City, IA
Microdystrophin	R1-16	Rabbit polyclonal antibody (1:400 dilution)	NovoPro Bioscience Inc. Shanghai, China
Laminin	Anti-laminin	Rabbit polyclonal antibody (1:200 dilution)	Sigma-Aldrich, St. Louis, MO
Laminin	2E8	Mouse monoclonal antibody, IgG2a (1:100 dilution)	Thermo Fisher Scientific, Hampton, NH

Secondary antibody	Supplier
Alexa Fluor 350 conjugated goat anti-mouse IgG2b (1:50 dilution)	Thermo Fisher Scientific, Hampton, NH
Alexa Fluor 647 conjugated goat anti-mouse IgG2a (1:100 dilution)	Thermo Fisher Scientific, Hampton, NH
Alexa Fluor 488 conjugated F(ab')2 goat anti-rabbit IgG (H+L) (1:100 dilution)	Thermo Fisher Scientific, Hampton, NH
Alexa Fluor 594 conjugated goat anti-mouse IgG1 (1:100 dilution)	Thermo Fisher Scientific, Hampton, NH
Fluorescein isothiocyanate conjugated goat anti-mouse IgM (1:100 dilution)	Jackson Immuno Research Labs, West Grove, PA

Figure S1. Dose response. A, Correlation between the AAV dose and AP expression in seven dogs (dogs #1-5, 11, and 12 in Table 1) that received the AAV-AP vector. Three muscles (biceps femoris, teres major, and latissimus dorsi) were examined in each injected dog except for one dog (dog #11) in which the teres major was not examined. **B,** Correlation between the AAV dose and uDys expression in ten dogs (dogs #6-10 and 13-17 in Table 1) that received the AAV-uDys vector. Three muscles (biceps femoris, teres major, and latissimus dorsi) were examined in each injected dog. **C,** Correlation between the AAV dose and transgene expression in all 17 AAV injected dogs. Each point represents one muscle from one dog. Best fit lines are calculated by simple linear regression.

Figure S1

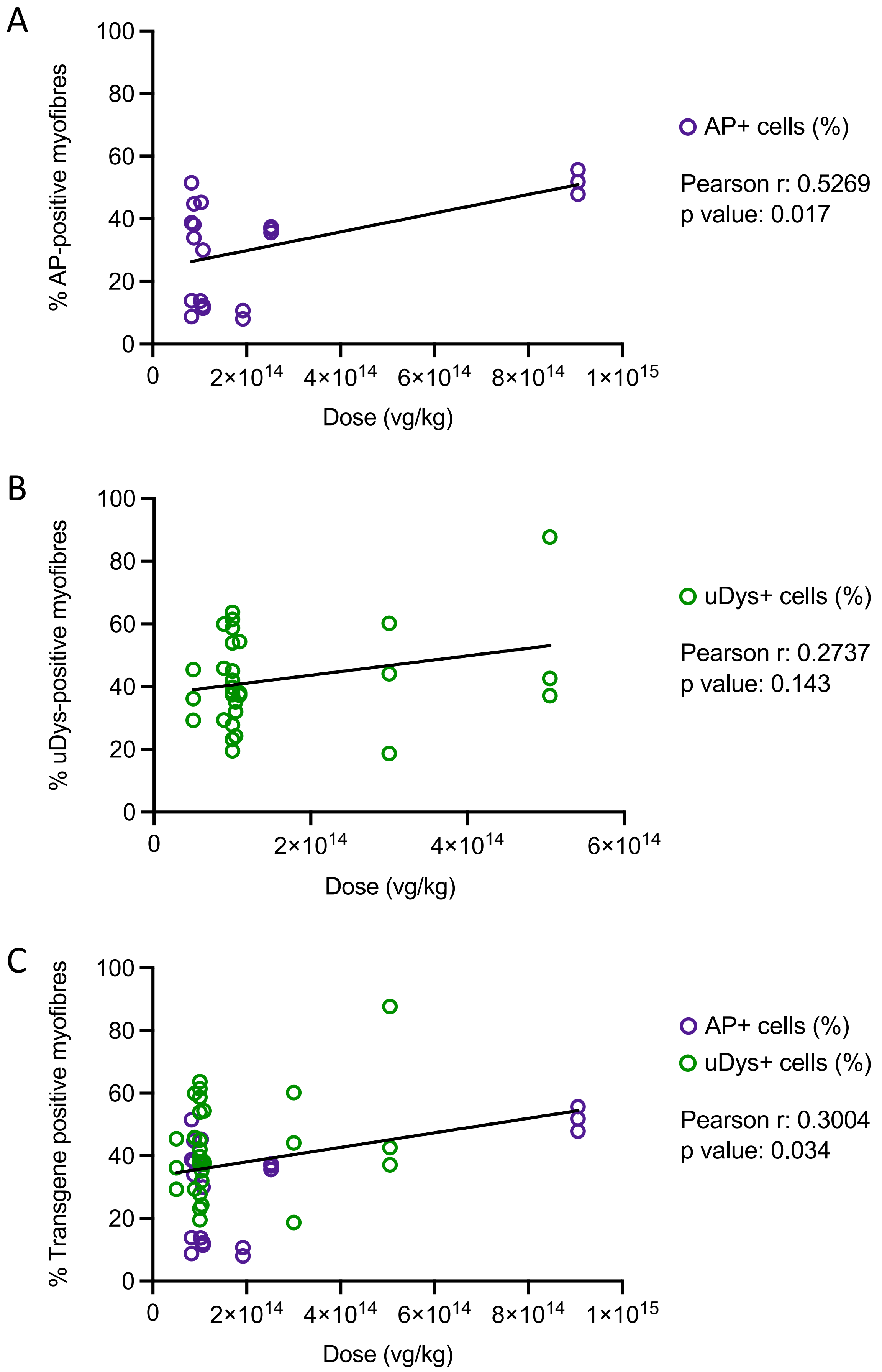
A
100
80
60
40
20
0
% AP-positive myofibres
0
2×10^14
4×10^14
6×10^14
8×10^14
1×10^15
Dose (vg/kg)
AP+ cells (%)
Pearson r: 0.5269
p value: 0.017
B
100
80
60
40
20
0
% uDys-positive myofibres
0
2×10^14
4×10^14
6×10^14
Dose (vg/kg)
uDys+ cells (%)
Pearson r: 0.2737
p value: 0.143
C
100
80
60
40
20
0
% Transgene positive myofibres
0
2×10^14
4×10^14
6×10^14
8×10^14
1×10^15
Dose (vg/kg)
AP+ cells (%)
uDys+ cells (%)
Pearson r: 0.3004
p value: 0.034

Figure S2. Biceps femoris, teres major, and latissimus dorsi showed mosaic transgene expression following systemic AAV injection. Representative photomicrographs of transgene expression in all 17 experimental dogs. **A,** AAV8 AP vector-injected dogs (Dog #1 to 5). **B,** AAV9 AP vector-injected dogs (Dog #11 and 12). **C,** AAV8 uDys vector-injected dogs (Dog #6 to 10). **D,** AAV9 uDys vector-injected dogs (Dog #13 to 17).

Figure S2A

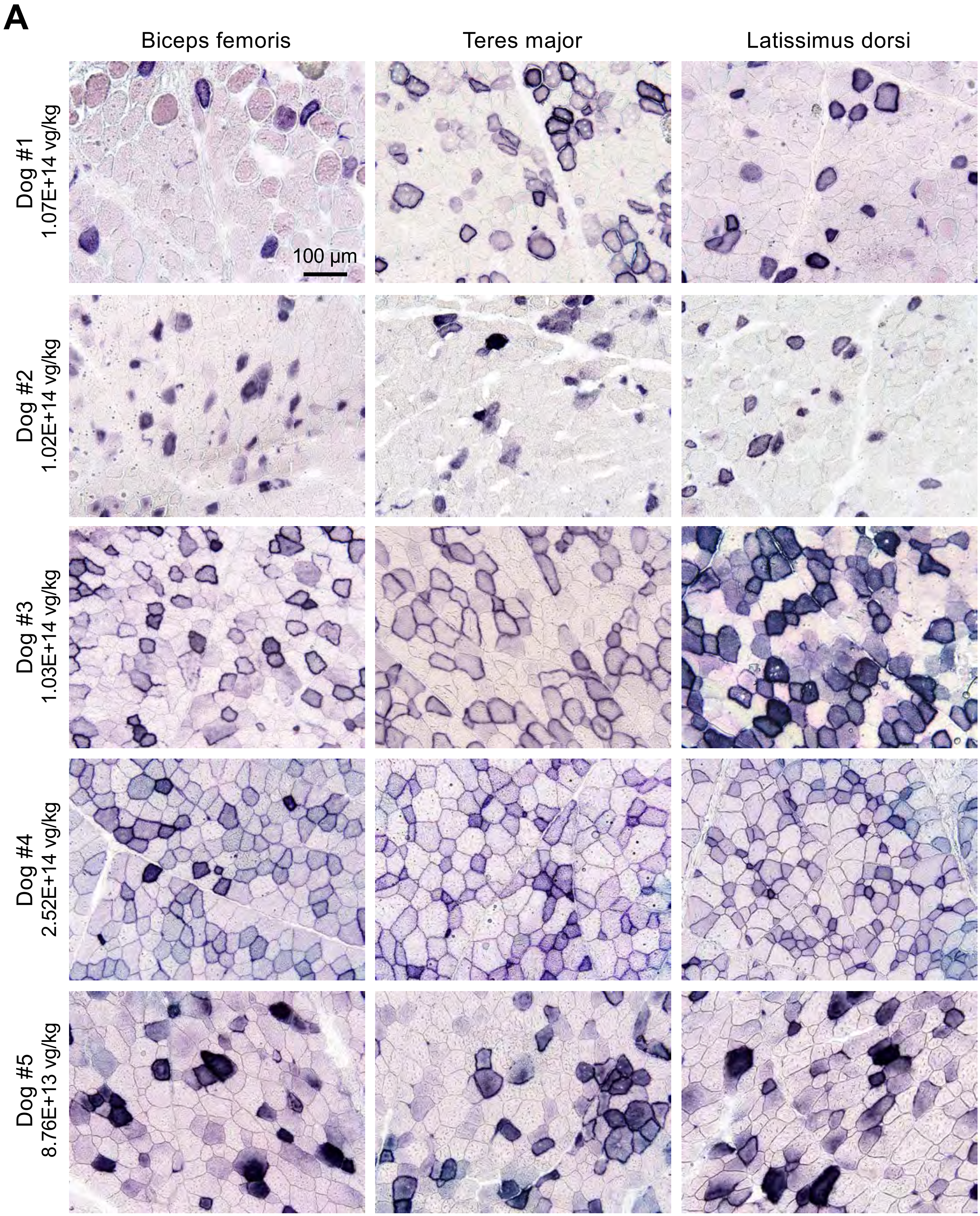
A
Biceps femoris
Teres major
Latissimus dorsi
Dog #1
1.07E+14 vg/kg
100 µm
Dog #2
1.02E+14 vg/kg
Dog #3
1.03E+14 vg/kg
Dog #4
2.52E+14 vg/kg
Dog #5
8.76E+13 vg/kg

Figure S2B

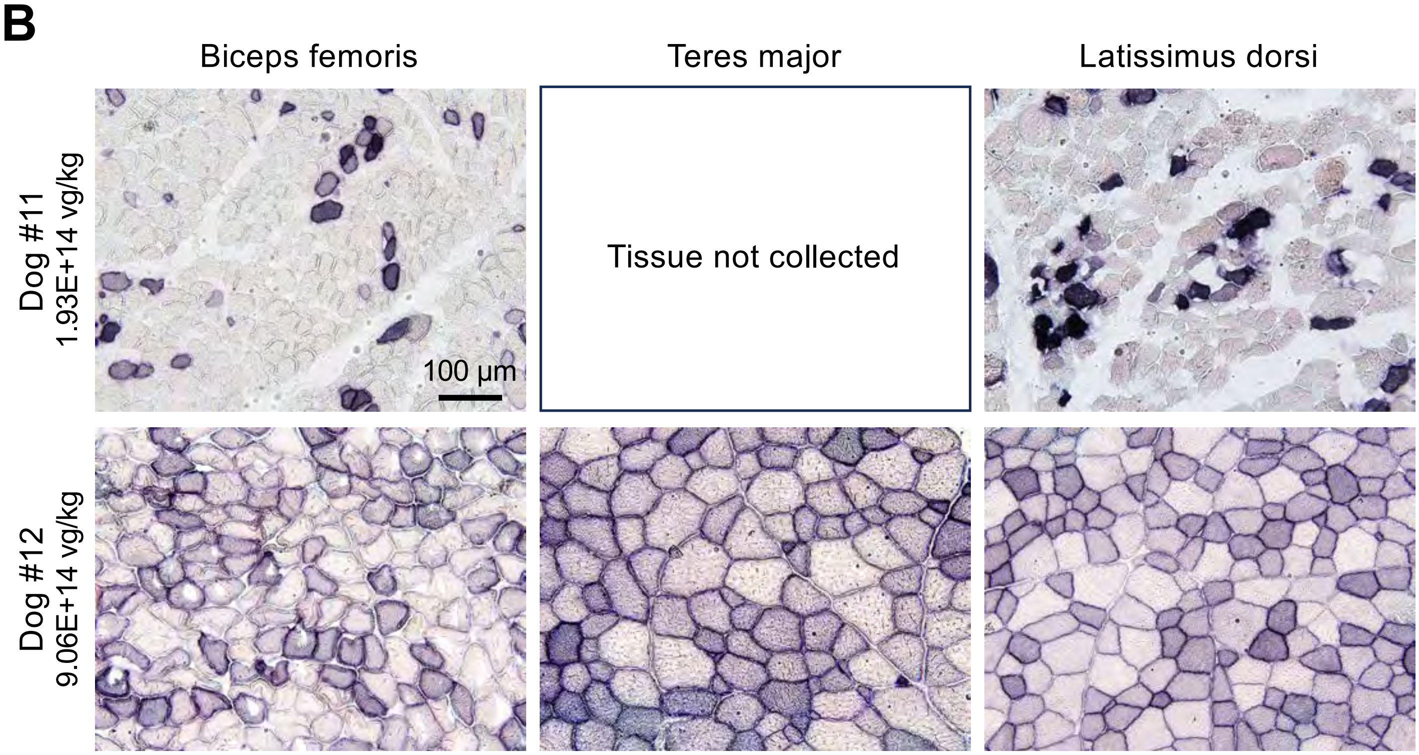
B
Biceps femoris
Teres major
Latissimus dorsi
Dog #11
1.93E+14 vg/kg
Dog #12
9.06E+14 vg/kg
100 µm
Tissue not collected

Figure S2C

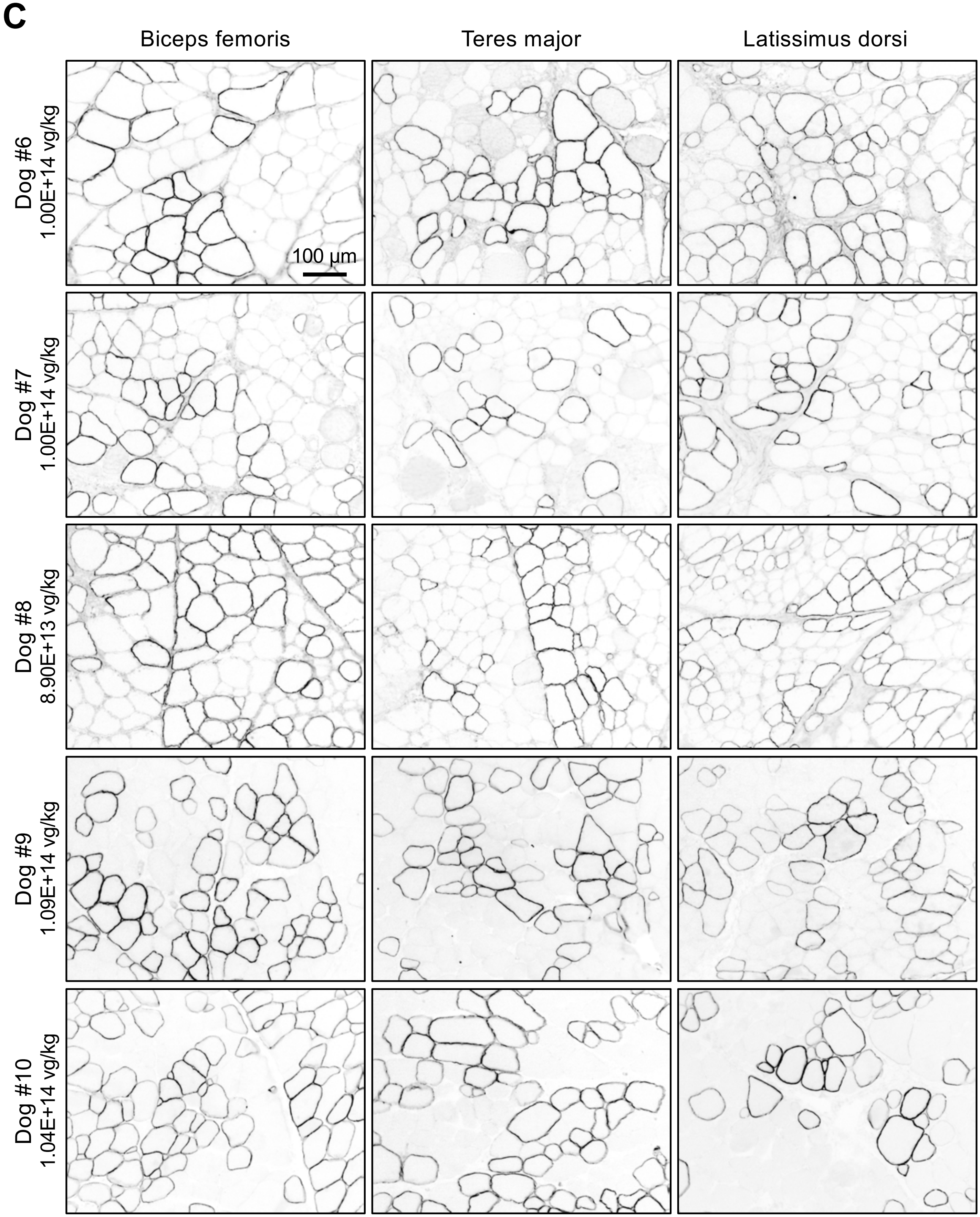
C
Biceps femoris
Teres major
Latissimus dorsi
Dog #6
1.00E+14 vg/kg
100 µm
Dog #7
1.00E+14 vg/kg
Dog #8
8.90E+13 vg/kg
Dog #9
1.09E+14 vg/kg
Dog #10
1.04E+14 vg/kg

Figure S2D

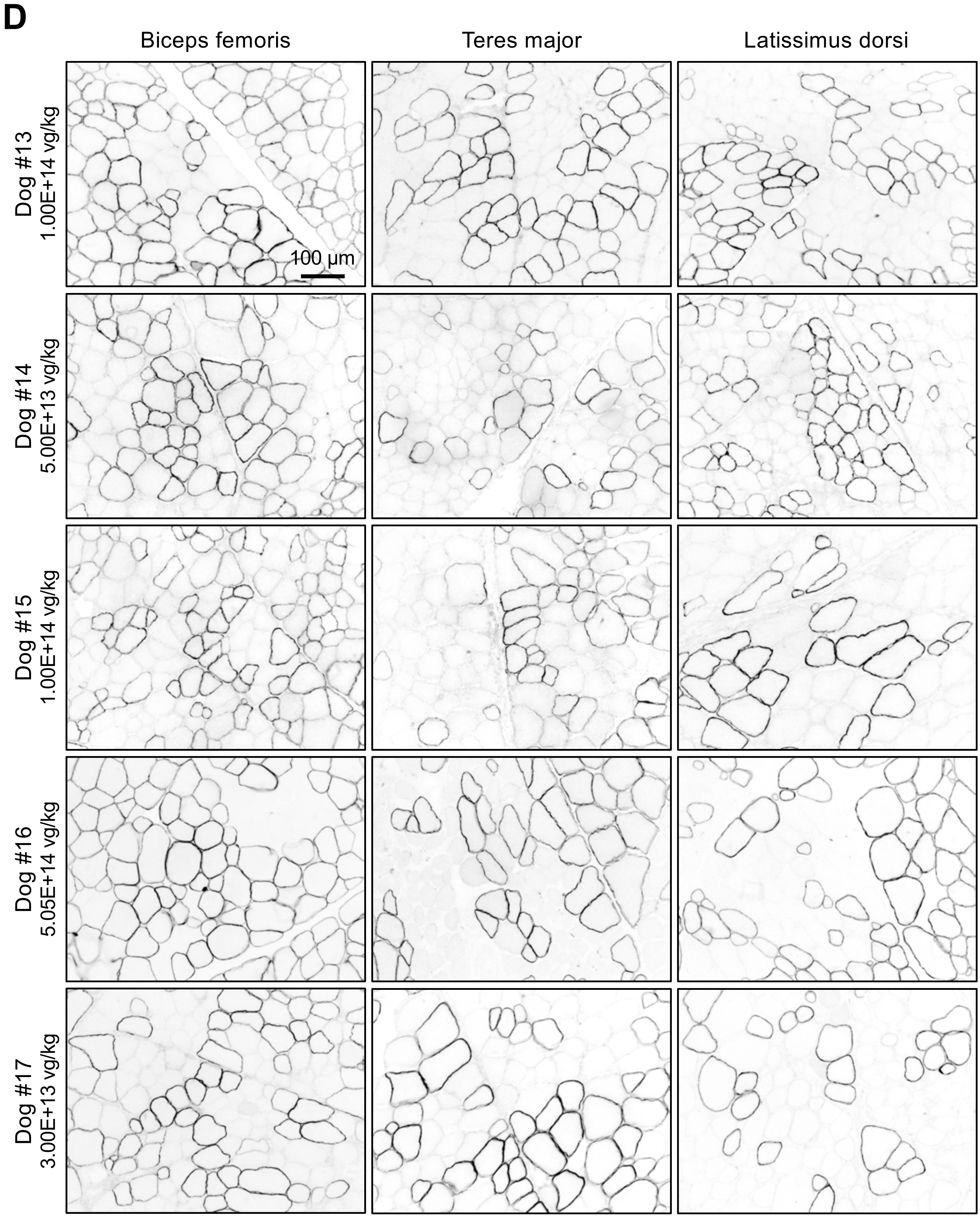
D
Biceps femoris
Teres major
Latissimus dorsi
Dog #13
1.00E+14 vg/kg
100 µm
Dog #14
5.00E+13 vg/kg
Dog #15
1.00E+14 vg/kg
Dog #16
5.05E+14 vg/kg
Dog #17
3.00E+13 vg/kg

Figure S3. AAV AP-injected affected and non-affected dogs showed similar fibre type distributions. A, Overall fibre type composition of the biceps femoris, teres major, and latissimus dorsi in four non-affected dogs (one normal and three carriers; dogs #3-5, and 12 in Table 1). **B,** Overall fibre type composition of the biceps femoris, teres major, and latissimus dorsi in three affected DMD dogs (dogs #1, 2, and 11 in Table 1). Please note, the teres major was not examined in dog #11. **C,** Comparison of overall fibre type distribution in affected and non-affected dogs. **D,** Comparison of fibre type distribution in the biceps femoris of affected and non-affected dogs. **E,** Comparison of fibre type distribution in the teres major of affected and non-affected dogs. **F,** Comparison of fibre type distribution in the latissimus dorsi of affected and non-affected dogs. ns, not significant, * $p < 0.05$, ** $p < 0.01$, *** $p < 0.001$, **** $p < 0.0001$.

Figure S3

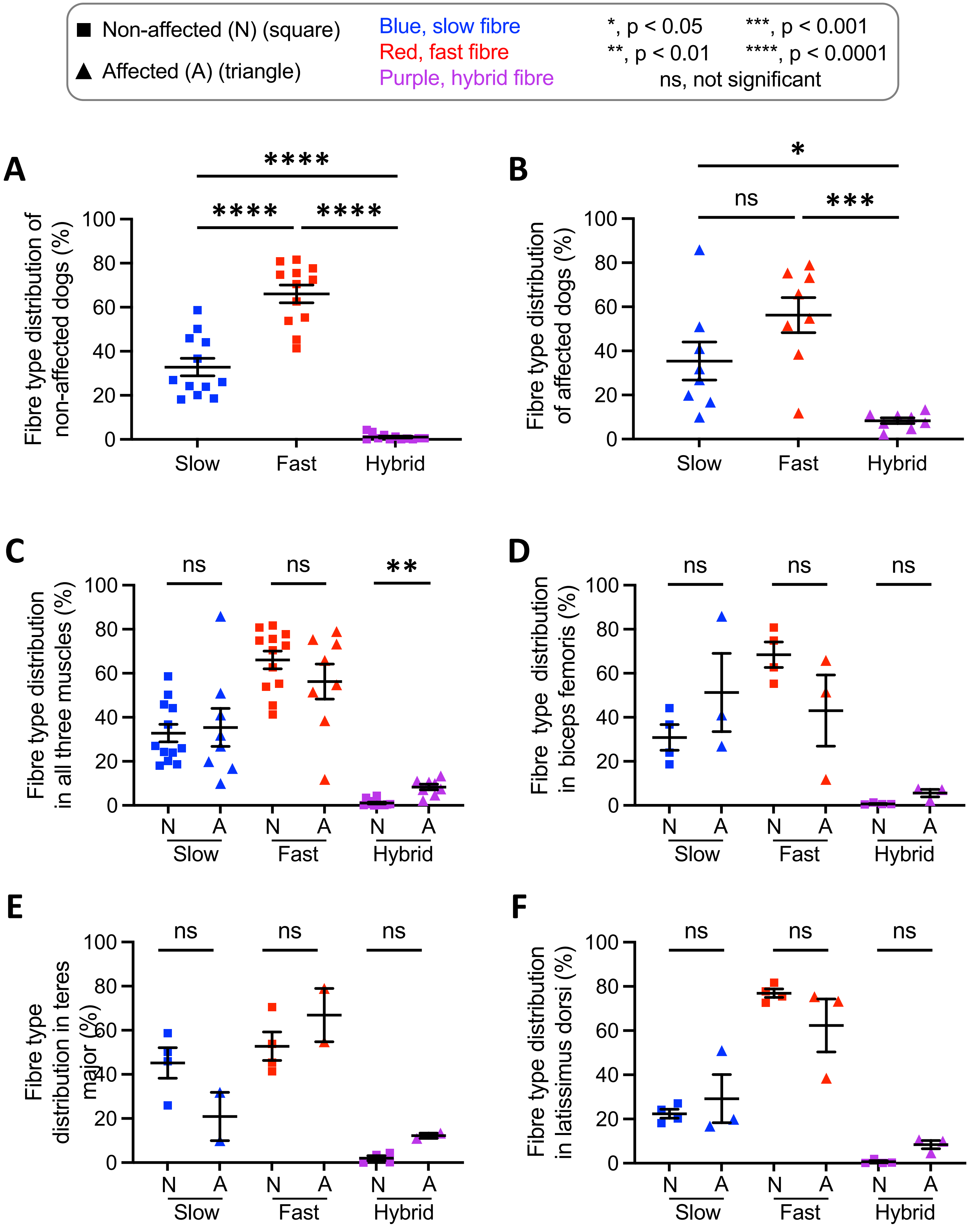

Non-affected (N) (square)
Affected (A) (triangle)
Blue, slow fibre
Red, fast fibre
Purple, hybrid fibre
*, p < 0.05
**, p < 0.01
***, p < 0.001
****, p < 0.0001
ns, not significant
A
Fibre type distribution of non-affected dogs (%)
Slow
Fast
Hybrid
****
B
Fibre type distribution of affected dogs (%)
ns
***
*
C
Fibre type distribution in all three muscles (%)
N
A
**
D
Fibre type distribution in biceps femoris (%)
E
Fibre type distribution in teres major (%)
F
Fibre type distribution in latissimus dorsi (%)

Figure S4. Fibre type distributions of uDys-positive and uDys-negative fibres were similar in ten affected dogs that received the AAV uDys vector. A, Comparison of fibre type distribution of uDys-positive and uDys-negative fibres in the biceps femoris. **B,** Comparison of fibre type distribution of uDys-positive and uDys-negative fibres in the teres major. **C,** Comparison of fibre type distribution of uDys-positive and uDys-negative fibres in the latissimus dorsi. ns, not significant, ** $p < 0.01$.

Figure S4

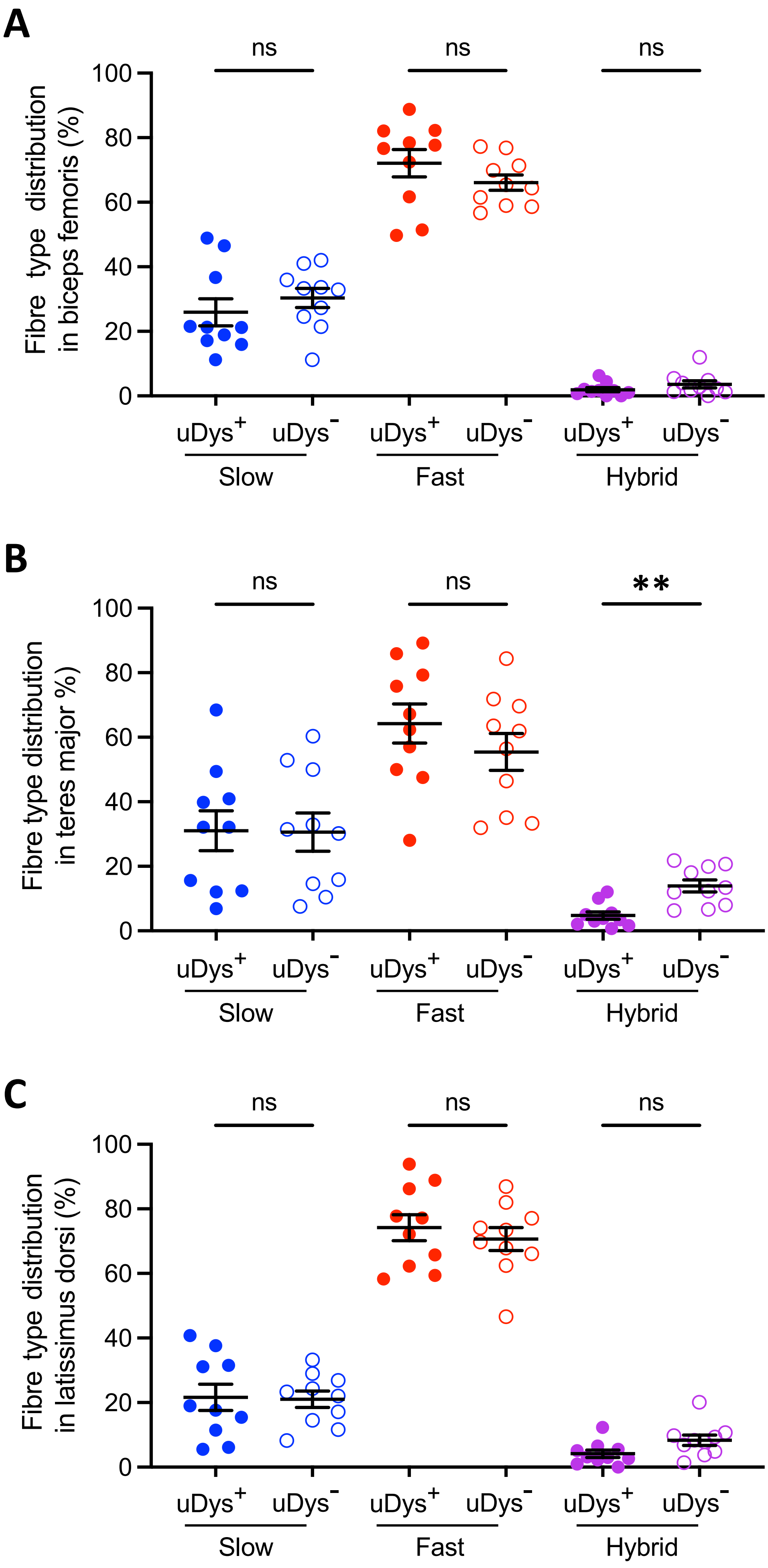

A
ns
ns
ns
Fibre type distribution in biceps femoris (%)
100
80
60
40
20
0
uDys+ uDys- uDys+ uDys- uDys+ uDys-
Slow
Fast
Hybrid
B
ns
ns
**
Fibre type distribution in teres major %)
100
80
60
40
20
0
uDys+ uDys- uDys+ uDys- uDys+ uDys-
Slow
Fast
Hybrid
C
ns
ns
ns
Fibre type distribution in latissimus dorsi (%)
100
80
60
40
20
0
uDys+ uDys- uDys+ uDys- uDys+ uDys-
Slow
Fast
Hybrid

Figure S5. Fibre size distributions in slow, fast, and hybrid fibres of the AP vector injected muscles. A, Mini-Feret diameter of AP-positive and AP-negative myofibres in slow, fast, and hybrid myofibres in AAV8 AP vector injected dogs. **B,** Cross-sectional area of AP-positive and AP-negative myofibres in slow, fast, and hybrid myofibres in AAV8 AP vector injected dogs. **C,** Mini-Feret diameter of AP-positive and AP-negative myofibres in slow, fast, and hybrid myofibres in AAV9 AP vector injected dogs. **D,** Cross-sectional area of AP-positive and AP-negative myofibres in slow, fast, and hybrid myofibres in AAV9 AP vector injected dogs. AAV8 AP-positive slow fibres, N=899; AAV8 AP-negative slow fibres, N=1,677; AAV8 AP-positive fast fibres, N=1,639; AAV8 AP-negative fast fibres, N=3,622; AAV8 AP-positive hybrid fibres, N=80; AAV8 AP-negative hybrid fibres, N=262. AAV9 AP-positive slow fibres, N=314; AAV9 AP-negative slow fibres, N=263; AAV9 AP-positive fast fibres, N=301; AAV9 AP-negative fast fibres, N=945; AAV9 AP-positive hybrid fibres, N=9; AAV9 AP-negative hybrid fibres, N=45.

Figure S5

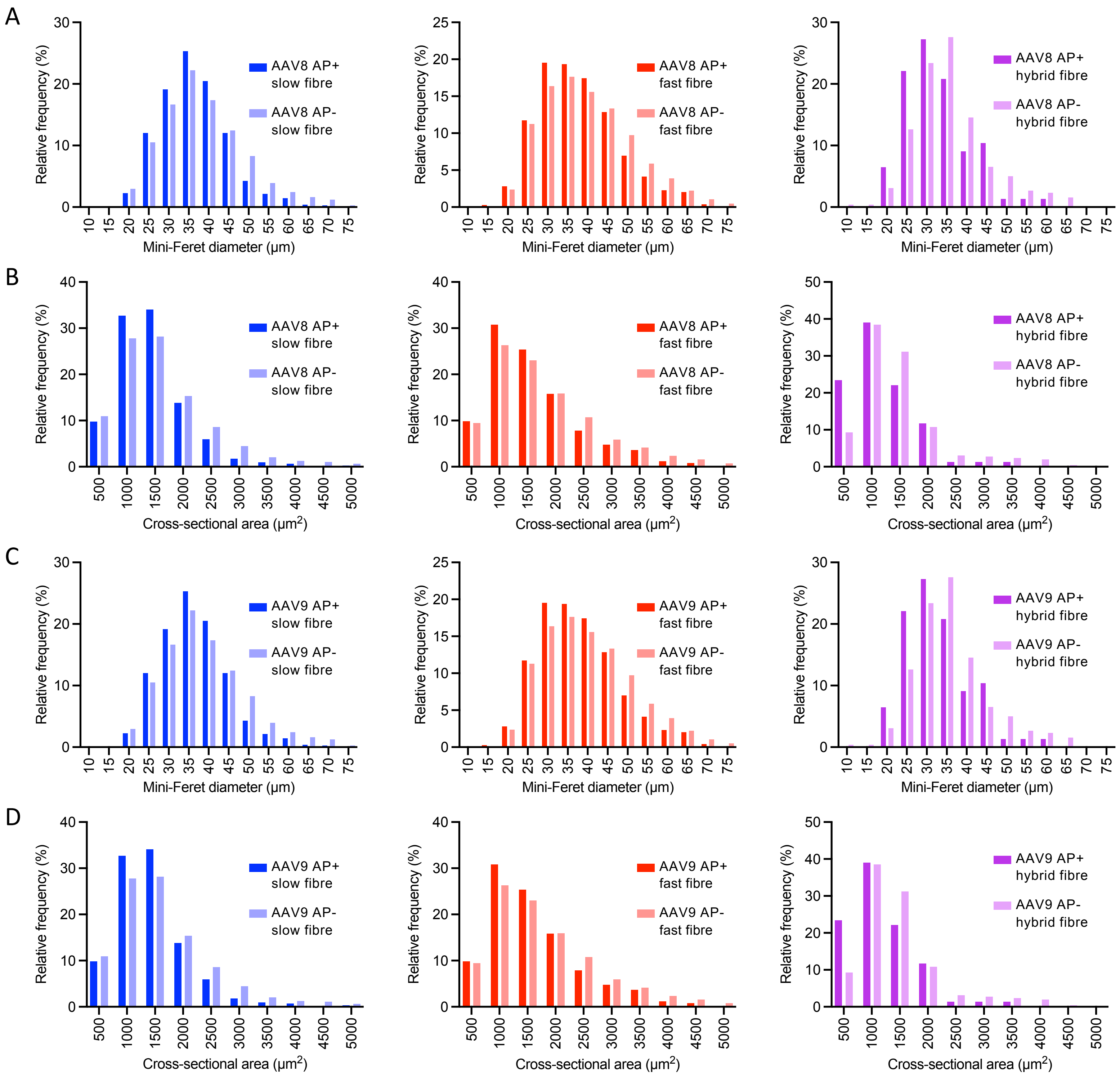

A
Relative frequency (%)
Mini-Feret diameter (μm)
AAV8 AP+ slow fibre
AAV8 AP- slow fibre
AAV8 AP+ fast fibre
AAV8 AP- fast fibre
AAV8 AP+ hybrid fibre
AAV8 AP- hybrid fibre
B
Relative frequency (%)
Cross-sectional area (μm2)
AAV8 AP+ slow fibre
AAV8 AP- slow fibre
AAV8 AP+ fast fibre
AAV8 AP- fast fibre
AAV8 AP+ hybrid fibre
AAV8 AP- hybrid fibre
C
Relative frequency (%)
Mini-Feret diameter (μm)
AAV9 AP+ slow fibre
AAV9 AP- slow fibre
AAV9 AP+ fast fibre
AAV9 AP- fast fibre
AAV9 AP+ hybrid fibre
AAV9 AP- hybrid fibre
D
Relative frequency (%)
Cross-sectional area (μm2)
AAV9 AP+ slow fibre
AAV9 AP- slow fibre
AAV9 AP+ fast fibre
AAV9 AP- fast fibre
AAV9 AP+ hybrid fibre
AAV9 AP- hybrid fibre

Figure S6. Evaluation of fibre size distribution in AP-positive and AP-negative myofibres following systemic injection of the AAV8 AP vector. A, Scatter plots and histogram graphs of the mini-Feret diameter (left panel) and cross-sectional area (right panel) of AP-positive and AP-negative slow, fast, and hybrid myofibres in AAV8 AP vector injected affected dogs. In scatter plots, data are presented as mean ± 95% confidence interval, and each point represents data from one myofibre. AP-positive slow fibres, N=176; AP-negative slow fibres, N=797; AP-positive fast fibres, N=198; AP-negative fast fibres, N=1,324; AP-positive hybrid fibres, N=55; AP-negative hybrid fibres, N=214. **B,** Scatter plots and histogram graphs of the mini-Feret diameter (left panel) and cross-sectional area (right panel) of AP-positive and AP-negative slow, fast, and hybrid myofibres in AAV8 AP vector injected carrier dogs. In scatter plots, data are presented as mean ± 95% confidence interval, and each point represents data from one myofibre. AP-positive slow fibres, N=723; AP-negative slow fibres, N=880; AP-positive fast fibres, N=1,441; AP-negative fast fibres, N=2,298; AP-positive hybrid fibres, N=25; AP-negative hybrid fibres, N=48.

Figure S6

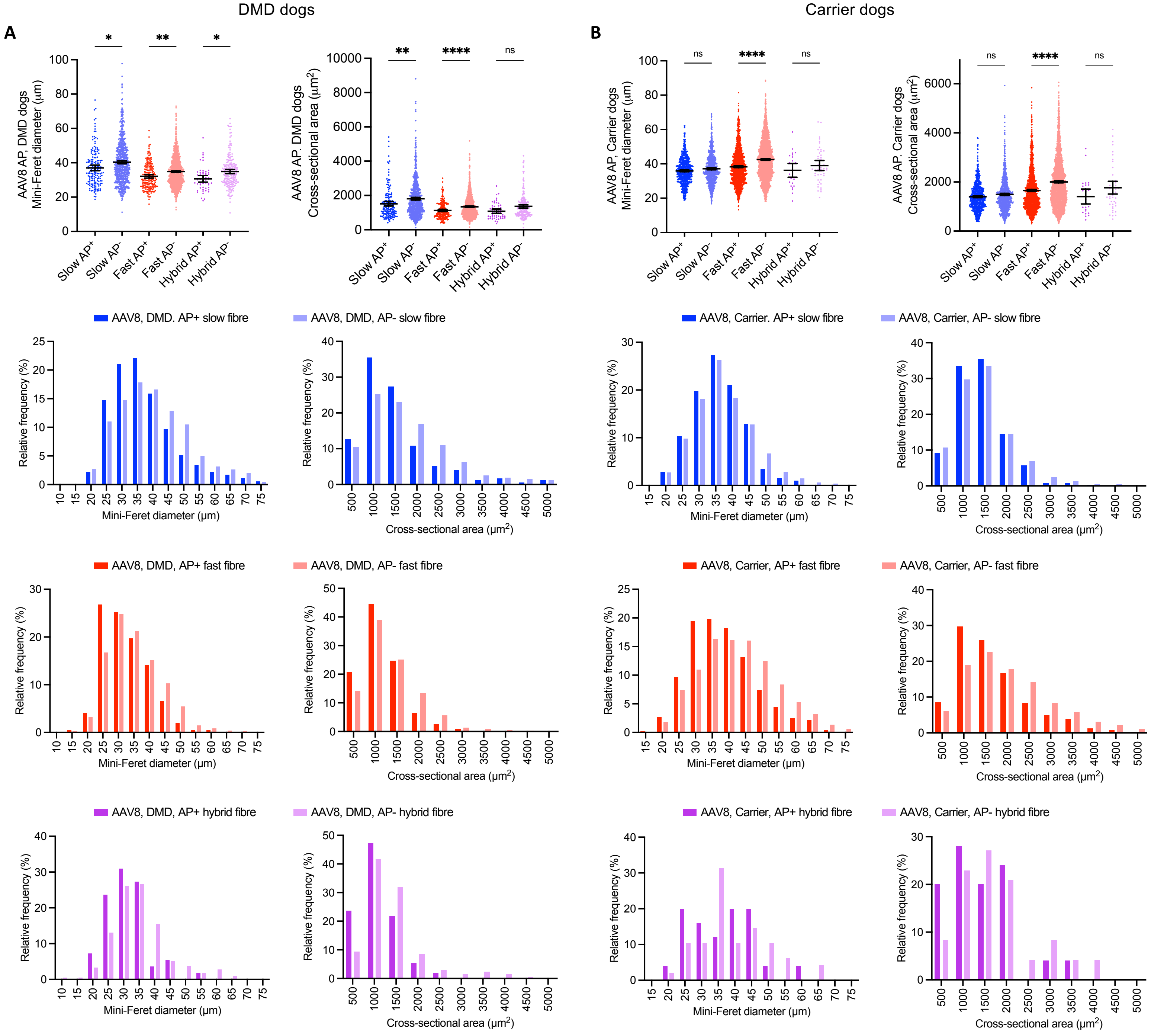

DMD dogs
Carrier dogs
A
B
AAV8 AP, DMD dogs Mini-Feret diameter (µm)
AAV8 AP. DMD dogs Cross-sectional area (µm2)
AAV8 AP, Carrier dogs Mini-Feret diameter (µm)
AAV8 AP. Carrier dogs Cross-sectional area (µm2)
Slow AP+
Slow AP-
Fast AP+
Fast AP-
Hybrid AP+
Hybrid AP-
AAV8, DMD. AP+ slow fibre
AAV8, DMD, AP- slow fibre
AAV8, Carrier. AP+ slow fibre
AAV8, Carrier, AP- slow fibre
AAV8, DMD, AP+ fast fibre
AAV8, DMD, AP- fast fibre
AAV8, Carrier, AP+ fast fibre
AAV8, Carrier, AP- fast fibre
AAV8, DMD, AP+ hybrid fibre
AAV8, DMD, AP- hybrid fibre
AAV8, Carrier, AP+ hybrid fibre
AAV8, Carrier, AP- hybrid fibre
Relative frequency (%)
Mini-Feret diameter (µm)
Cross-sectional area (µm2)

Figure S7. Evaluation of fibre size distribution in AP-positive and AP-negative myofibres following systemic injection of the AAV9 AP vector. A, Scatter plots and histogram graphs of the mini-Feret diameter (left panel) and cross-sectional area (right panel) of AP-positive and AP-negative slow, fast, and hybrid myofibres in the AAV9 AP vector injected affected dog. In scatter plots, data are presented as mean ± 95% confidence interval, and each point represents data from one myofibre. AP-positive slow fibres, N=35; AP-negative slow fibres, N=168; AP-positive fast fibres, N=37; AP-negative fast fibres, N=562; AP-positive hybrid fibres, N=7; AP-negative hybrid fibres, N=45. **B,** Scatter plots and histogram graphs of the mini-Feret diameter (left panel) and cross-sectional area (right panel) of AP-positive and AP-negative slow, fast, and hybrid myofibres in the AAV9 AP vector injected normal dog. In scatter plots, data are presented as mean ± 95% confidence interval, and each point represents data from one myofibre. AP-positive slow fibres, N=276; AP-negative slow fibres, N=95; AP-positive fast fibres, N=264; AP-negative fast fibres, N=383; AP-positive hybrid fibres, N=2; AP-negative hybrid fibres, N=0.

Figure S7

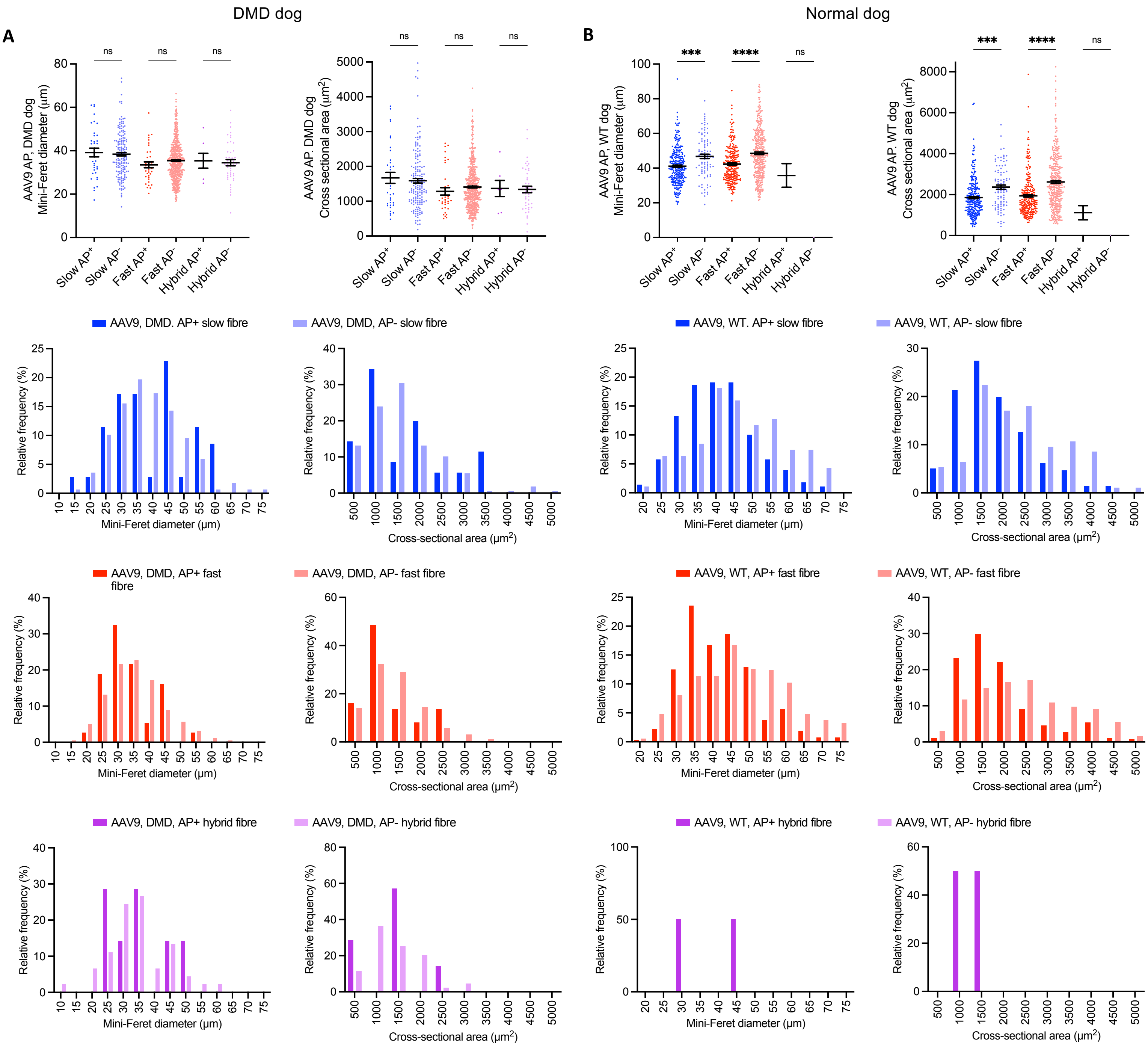

DMD dog
Normal dog
A
B
ns
***
****
AAV9 AP, DMD dog Mini-Feret diameter (µm)
AAV9 AP. DMD dog Cross sectional area (µm²)
AAV9 AP, WT dog Mini-Feret diameter (µm)
AAV9 AP. WT dog Cross sectional area (µm²)
Slow AP+
Slow AP-
Fast AP+
Fast AP-
Hybrid AP+
Hybrid AP-
AAV9, DMD. AP+ slow fibre
AAV9, DMD, AP- slow fibre
AAV9, WT. AP+ slow fibre
AAV9, WT, AP- slow fibre
AAV9, DMD, AP+ fast fibre
AAV9, DMD, AP- fast fibre
AAV9, WT, AP+ fast fibre
AAV9, WT, AP- fast fibre
AAV9, DMD, AP+ hybrid fibre
AAV9, DMD, AP- hybrid fibre
AAV9, WT, AP+ hybrid fibre
AAV9, WT, AP- hybrid fibre
Relative frequency (%)
Mini-Feret diameter (µm)
Cross-sectional area (µm²)

Figure S8. Evaluation of fibre size distribution in slow, fast, and hybrid myofibres of normal and affected dogs. Scatter plots and histogram graphs of the mini-Feret diameter (left panel) and cross-sectional area (right panel) in slow, fast, and hybrid myofibres of three normal (WT) and three affected (DMD) dogs (dog #18-23 in Table 1). In scatter plots, data are presented as mean ± 95% confidence interval, and each point represents data from one myofibre. Slow fibres of affected dogs, N=1,903; Slow fibres of normal dogs, N=1,819; Fast fibres of affected dogs, N=2,421; Fast fibres of normal dogs, N=2,665; Hybrid fibres of affected dogs, N=703; Hybrid fibres of normal dogs, N=395.

Figure S8

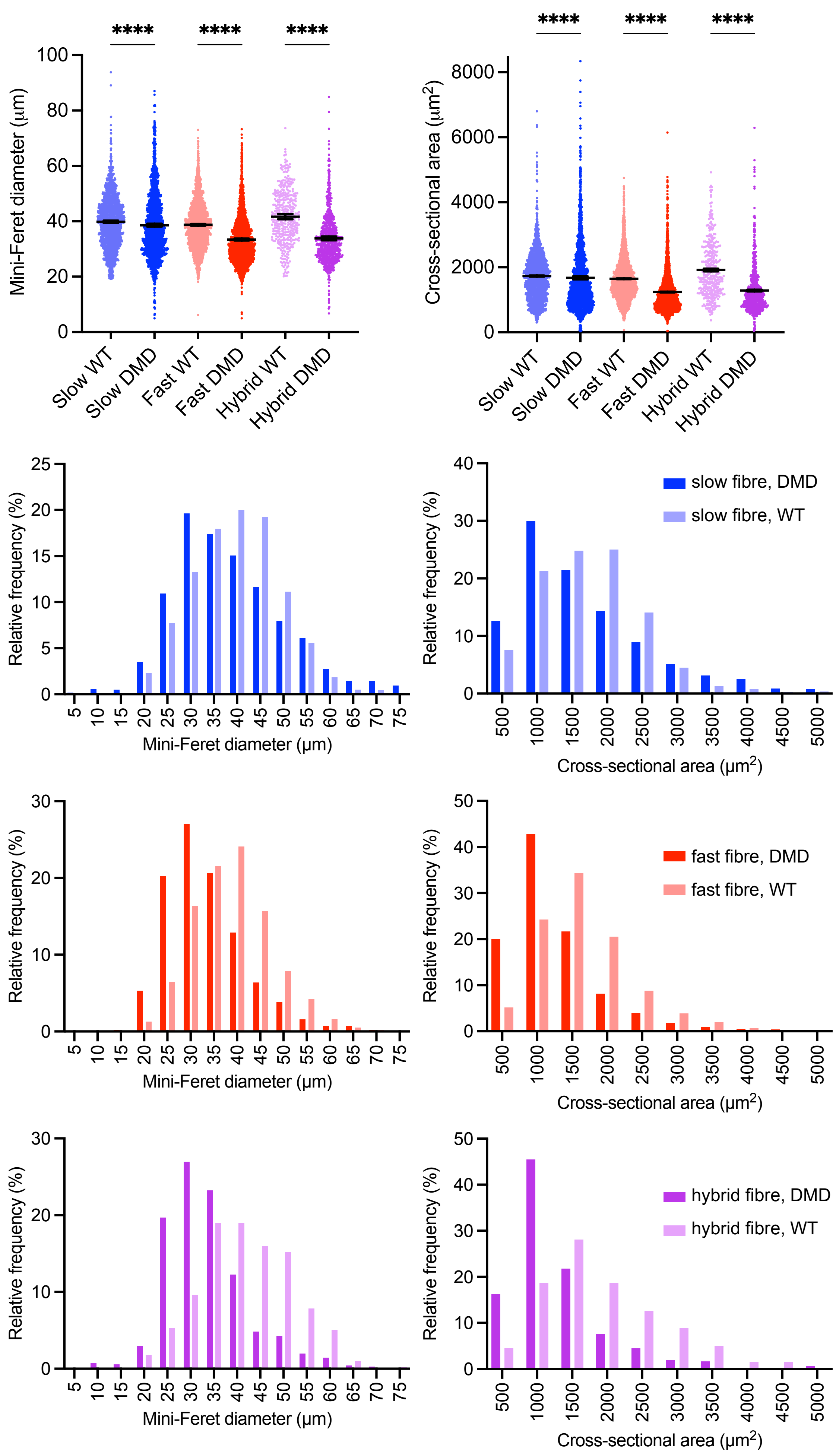

****
Mini-Feret diameter (µm)
Slow WT
Slow DMD
Fast WT
Fast DMD
Hybrid WT
Hybrid DMD
Cross-sectional area (µm^2)
Relative frequency (%)
slow fibre, DMD
slow fibre, WT
fast fibre, DMD
fast fibre, WT
hybrid fibre, DMD
hybrid fibre, WT

Figure S9. Fibre size distributions in slow, fast, and hybrid fibres of the uDys vector injected muscles. A, Mini-Feret diameter of uDys-positive and uDys-negative slow, fast, and hybrid myofibres in AAV8 uDys vector injected dogs. **B,** Cross-sectional area of uDys-positive and uDys-negative slow, fast, and hybrid myofibres in AAV8 uDys vector injected dogs. **C,** Mini-Feret diameter of uDys-positive and uDys-negative slow, fast, and hybrid myofibres in AAV9 uDys vector injected dogs. **D,** Cross-sectional area of uDys-positive and uDys-negative slow, fast, and hybrid myofibres in AAV9 uDys vector injected dogs. AAV8 uDys-positive slow fibres, N=946; AAV8 uDys-negative slow fibres, N=1,261; AAV8 uDys-positive fast fibres, N=1,762; AAV8 uDys-negative fast fibres, N=2,870; AAV8 uDys-positive hybrid fibres, N=109; AAV8 uDys-negative hybrid fibres, N=444. AAV9 uDys-positive slow fibres, N=573; AAV9 uDys-negative slow fibres, N=908; AAV9 uDys-positive fast fibres, N=2,205; AAV9 uDys-negative fast fibres, N=2,293; AAV9 uDys-positive hybrid fibres, N=78; AAV9 uDys-negative hybrid fibres, N=329.

Figure S9

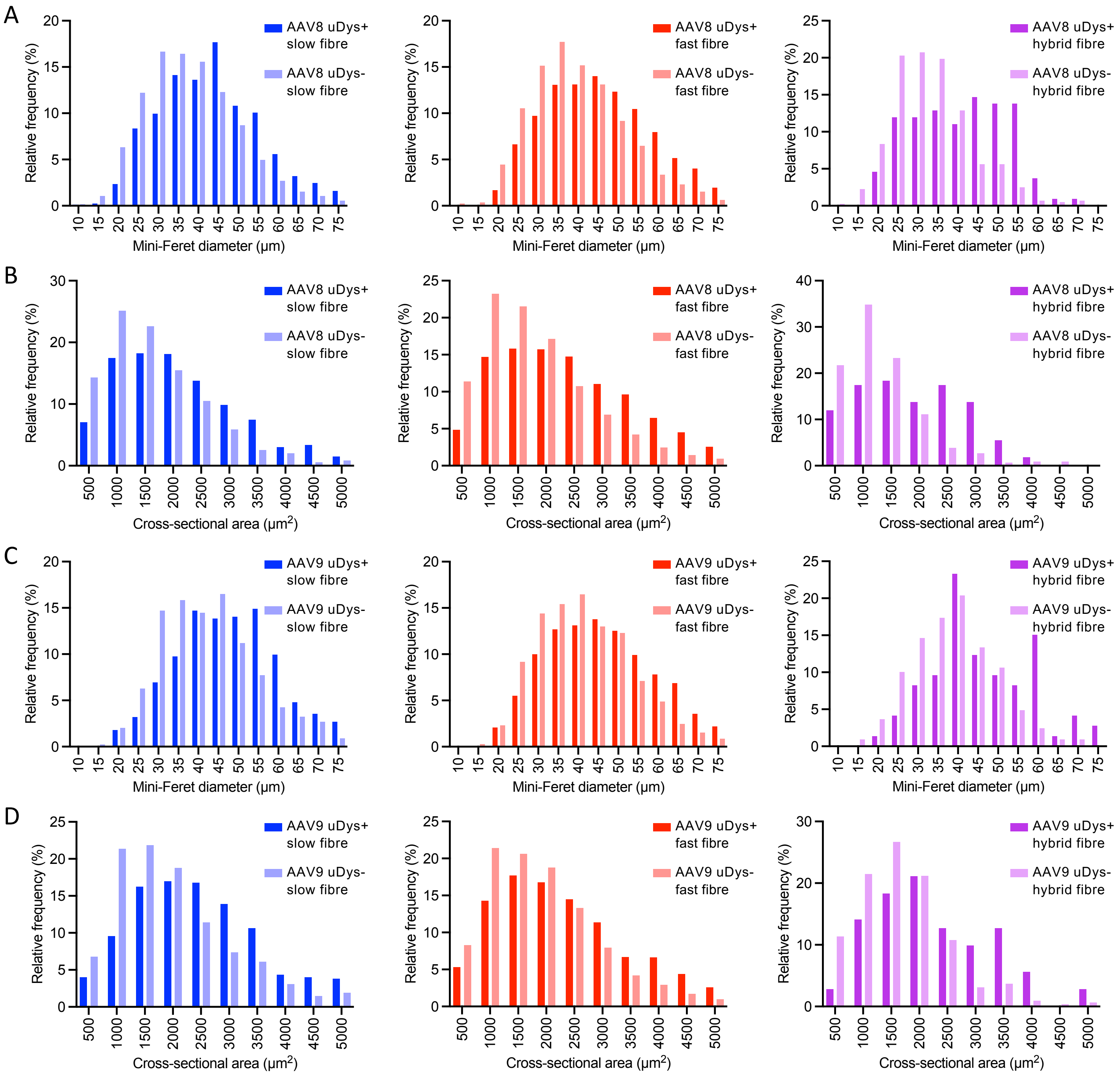

A
Relative frequency (%)
Mini-Feret diameter (µm)
AAV8 uDys+ slow fibre
AAV8 uDys- slow fibre
AAV8 uDys+ fast fibre
AAV8 uDys- fast fibre
AAV8 uDys+ hybrid fibre
AAV8 uDys- hybrid fibre
B
Relative frequency (%)
Cross-sectional area (µm2)
AAV8 uDys+ slow fibre
AAV8 uDys- slow fibre
AAV8 uDys+ fast fibre
AAV8 uDys- fast fibre
AAV8 uDys+ hybrid fibre
AAV8 uDys- hybrid fibre
C
Relative frequency (%)
Mini-Feret diameter (µm)
AAV9 uDys+ slow fibre
AAV9 uDys- slow fibre
AAV9 uDys+ fast fibre
AAV9 uDys- fast fibre
AAV9 uDys+ hybrid fibre
AAV9 uDys- hybrid fibre
D
Relative frequency (%)
Cross-sectional area (µm2)
AAV9 uDys+ slow fibre
AAV9 uDys- slow fibre
AAV9 uDys+ fast fibre
AAV9 uDys- fast fibre
AAV9 uDys+ hybrid fibre
AAV9 uDys- hybrid fibre

Figure S10. Evaluation of fibre size distribution in uDys-positive and uDys-negative myofibres following systemic injection of the AAV9 uDys vector. A, Scatter plots and histogram graphs of the mini-Feret diameter (left panel) and cross-sectional area (right panel) of uDys-positive and uDys-negative slow, fast, and hybrid myofibres in an affected dog that received 5 x 10^{13} vg/kg of the AAV9 uDys vector. In scatter plots, data are presented as mean ± 95% confidence interval, and each point represents data from one myofibre. uDys-positive slow fibres, N=96; uDys-negative slow fibres, N=245; uDys-positive fast fibres, N=398; uDys-negative fast fibres, N=573; uDys-positive hybrid fibres, N=1; uDys-negative hybrid fibres, N=37. **B,** Scatter plots and histogram graphs of the mini-Feret diameter (left panel) and cross-sectional area (right panel) of uDys-positive and uDys-negative slow, fast, and hybrid myofibres in two affected dogs that received 1 x 10^{14} vg/kg of the AAV9 uDys vector. In scatter plots, data are presented as mean ± 95% confidence interval, and each point represents data from one myofibre. uDys-positive slow fibres, N=296; uDys-negative slow fibres, N=430; uDys-positive fast fibres, N=950; uDys-negative fast fibres, N=902; uDys-positive hybrid fibres, N=41; uDys-negative hybrid fibres, N=191. **C,** Scatter plots and histogram graphs of the mini-Feret diameter (left panel) and cross-sectional area (right panel) of uDys-positive and uDys-negative slow, fast, and hybrid myofibres in an affected dog that received 3 x 10^{14} vg/kg of the AAV9 uDys vector. In scatter plots, data are presented as mean ± 95% confidence interval, and each point represents data from one myofibre. uDys-positive slow fibres, N=79; uDys-negative slow fibres, N=139; uDys-positive fast fibres, N=417; uDys-negative fast fibres, N=492; uDys-positive hybrid fibres, N=7; uDys-negative hybrid fibres, N=41. **D,** Scatter plots and histogram graphs of the mini-Feret diameter (left panel) and cross-sectional area (right panel) of uDys-positive and uDys-negative slow, fast, and hybrid myofibres in an affected dog that received 5 x 10^{14} vg/kg of the AAV9 uDys vector. In scatter plots, data are presented as mean ± 95% confidence interval, and each point represents data from one myofibre. uDys-positive slow fibres, N=102; uDys-

negative slow fibres, N=94; uDys-positive fast fibres, N=440; uDys-negative fast fibres, N=326; uDys-positive hybrid fibres, N=29; uDys-negative hybrid fibres, N=60.

Figure S10

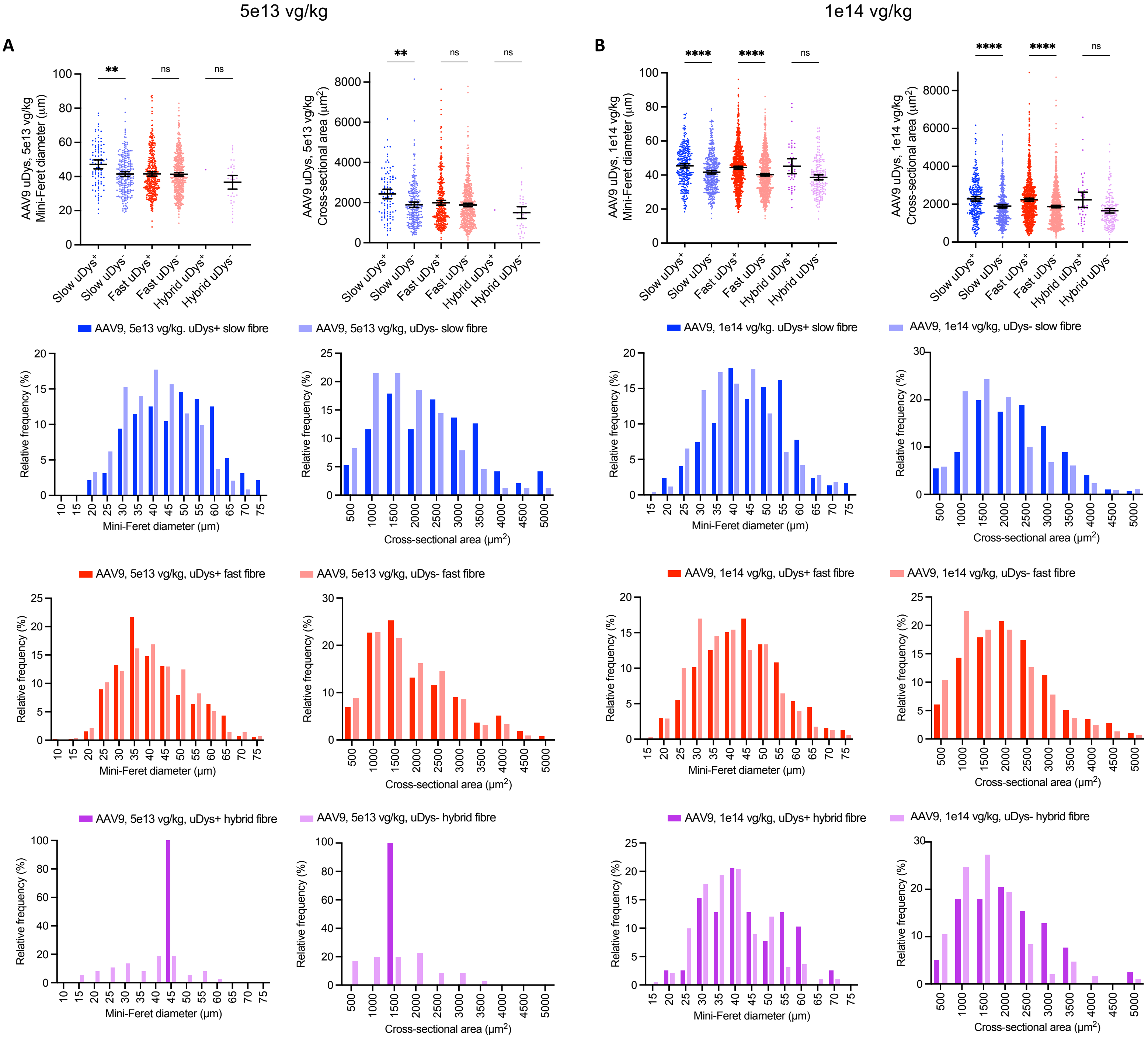

5e13 vg/kg
1e14 vg/kg
A
B
**
****
ns
AAV9 uDys, 5e13 vg/kg
Mini-Feret diameter (µm)
Cross-sectional area (µm2)
AAV9 uDys, 1e14 vg/kg
Slow uDys+
Slow uDys-
Fast uDys+
Fast uDys-
Hybrid uDys+
Hybrid uDys-
AAV9, 5e13 vg/kg. uDys+ slow fibre
AAV9, 5e13 vg/kg, uDys- slow fibre
AAV9, 1e14 vg/kg. uDys+ slow fibre
AAV9, 1e14 vg/kg, uDys- slow fibre
AAV9, 5e13 vg/kg, uDys+ fast fibre
AAV9, 5e13 vg/kg, uDys- fast fibre
AAV9, 1e14 vg/kg, uDys+ fast fibre
AAV9, 1e14 vg/kg, uDys- fast fibre
AAV9, 5e13 vg/kg, uDys+ hybrid fibre
AAV9, 5e13 vg/kg, uDys- hybrid fibre
AAV9, 1e14 vg/kg, uDys+ hybrid fibre
AAV9, 1e14 vg/kg, uDys- hybrid fibre
Relative frequency (%)
Mini-Feret diameter (µm)
Cross-sectional area (µm2)

Figure S10

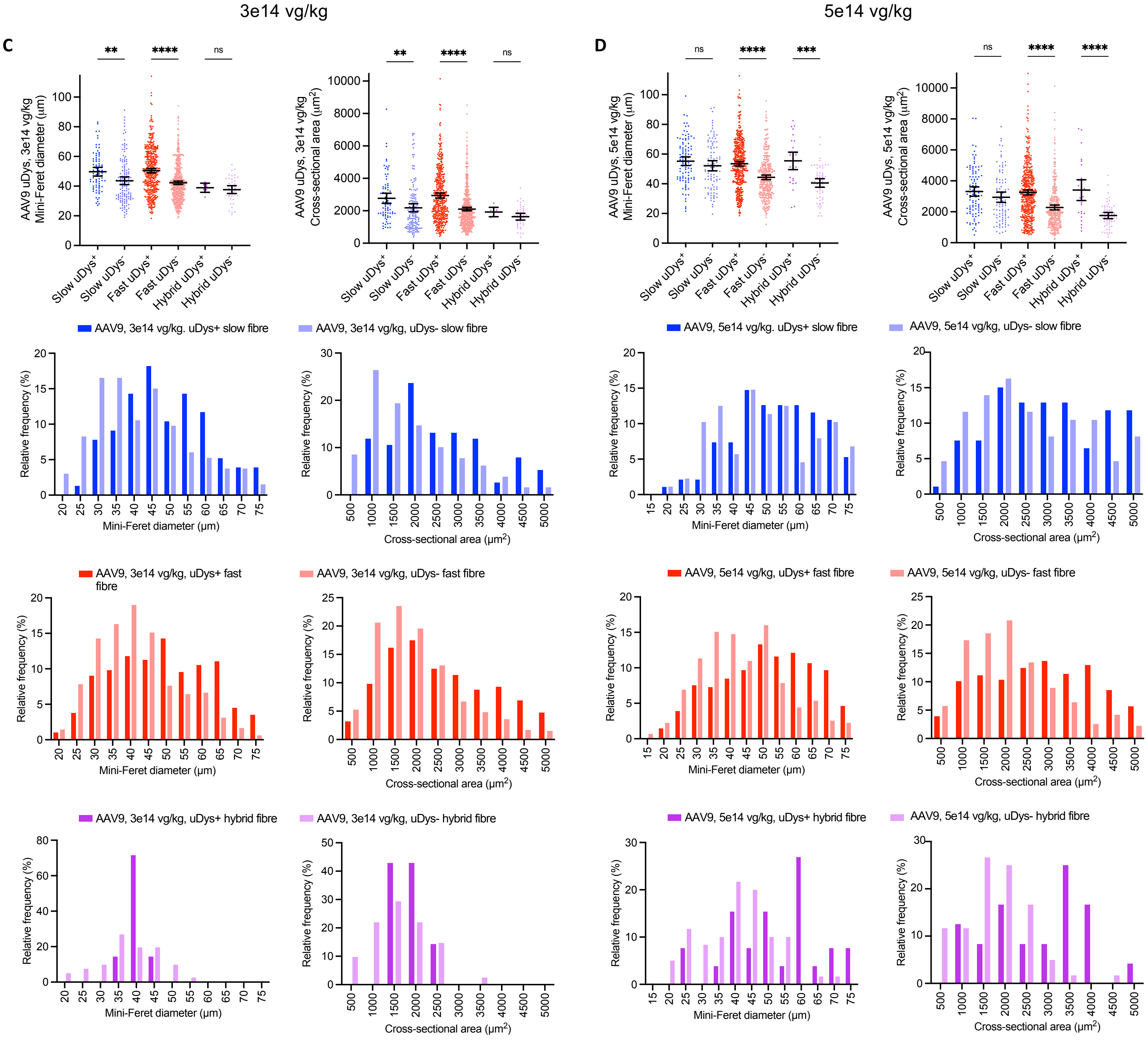
3e14 vg/kg
5e14 vg/kg
C
D
**
****
ns
***
AAV9 uDys, 3e14 vg/kg Mini-Feret diameter (µm)
AAV9 uDys, 3e14 vg/kg Cross-sectional area (µm2)
AAV9 uDys, 5e14 vg/kg Mini-Feret diameter (µm)
AAV9 uDys, 5e14 vg/kg Cross-sectional area (µm2)
Slow uDys+
Slow uDys-
Fast uDys+
Fast uDys-
Hybrid uDys+
Hybrid uDys-
AAV9, 3e14 vg/kg. uDys+ slow fibre
AAV9, 3e14 vg/kg, uDys- slow fibre
AAV9, 5e14 vg/kg. uDys+ slow fibre
AAV9, 5e14 vg/kg, uDys- slow fibre
AAV9, 3e14 vg/kg, uDys+ fast fibre
AAV9, 3e14 vg/kg, uDys- fast fibre
AAV9, 5e14 vg/kg, uDys+ fast fibre
AAV9, 5e14 vg/kg, uDys- fast fibre
AAV9, 3e14 vg/kg, uDys+ hybrid fibre
AAV9, 3e14 vg/kg, uDys- hybrid fibre
AAV9, 5e14 vg/kg, uDys+ hybrid fibre
AAV9, 5e14 vg/kg, uDys- hybrid fibre
Relative frequency (%)
Mini-Feret diameter (µm)
Cross-sectional area (µm2)
